# Supplementary material for: Siamese foundation models for crystal structure prediction
Source: Nat Commun. 2026 May 6;17:6103. doi: 10.1038/s41467-026-72362-3 (PMC13358074; doi:10.1038/s41467-026-72362-3)
Supplement: Supplementary file 1 — Supplementary Information: Siamese Foundation Models for Crystal Structure Prediction [file 41467_2026_72362_MOESM1_ESM.pdf]

Supplementary Information: Siamese Foundation Models for Crystal Structure Prediction

Liming Wu[1,2,3 †], Wenbing Huang[1,2,3 †, ✉], Rui Jiao[4,5], Jianxing Huang[6], Liwei Liu[6], Yipeng Zhou[6], Hao Sun[1,2,3], Yang Liu[4,5], Fuchun Sun[4], Yuxiang Ren[7, ✉], and Ji-Rong Wen[1,2,3, ✉]

[1]Gaoling School of Artificial Intelligence, Renmin University of China, Beijing, China
[2]Beijing Key Laboratory of Research on Large Models and Intelligent Governance
[3]Engineering Research Center of Next-Generation Intelligent Search and Recommendation, MOE
[4]Department of Computer Science and Technology, Tsinghua University, Beijing, China
[5]Institute for AI Industry Research, Tsinghua University, Beijing, China
[6]Advanced Computing and Storage Lab, Huawei Technologies, Shanghai, China
[7]School of Intelligence Science and Technology, Nanjing University, Suzhou, China
[†]These authors contributed equally: Liming Wu, Wenbing Huang
[✉]Correspondence should be addressed to: hwenbing@ruc.edu.cn; renyuxiang@nju.edu.cn; jrwen@ruc.edu.cn

Contents

A Preliminaries **2**
A.1 Diffusion Models 2
A.2 Equivariance and Invariance 2
B Theoretical Analysis **2**
B.1 Invariance of the learned Node Features 3
B.2 Equivariance of the Noise Output 3
B.3 Derivation of the Intermediate Energy Prediction Loss 3
C Downstream Datasets Introduction **4**
D Implementation Details **4**
D.1 Hyperparameters of DAO 4
D.2 Hyperparameters of Pretrained CSP Baselines 5
D.3 Configurations of Finetuning 5
E Extended Experimental Results **6**
E.1 Deeper Analysis of Structure Generation 6
E.1.1 Ablations on Two-Stage Pretraining 6
E.1.2 Ablations on Energy Relaxation Threshold 7
E.1.3 20-shot Results 8
E.2 Accurate Crystal Property Prediction via the Finetuned DAO-P 8
E.3 Additional Results on Superconductor Validation 10

E.3.1 Joint Superconducting Property Prediction and Structure Generation 11
E.3.2 DFT Results for Three Real-World Superconductors 12

F Visualization **13**
F.1 Visualization of Generated Polymorphs 13
F.2 Visualization of the Diffusion Process 13

G Comparison with DiffCSP and MatterGen **14**
G.1 Comparative Analysis with DiffCSP 14
G.2 Comparative Analysis with MatterGen 15

A Preliminaries

A.1 Diffusion Models

The diffusion process unfolds in two stages: a forward pass that adds noise to the data and a backward pass focused on denoising. During the forward process, noise is progressively added to the original data sample until it conforms to a predefined distribution at timestep T, commonly chosen to be a standard normal distribution. Denoising (the backward process) often requires the model to predict the noise introduced at each timestep, as in DDPM [1]. Alternatively, methods like Score-Matching [2] aim to predict the gradient of the log-likelihood, also known as the score s_θ. When utilizing diffusion models for the generation of geometric objects, particularly those in three dimensions, it is imperative to incorporate knowledge of the inherent symmetry present in the data to avoid generating unrealistic or physically implausible structures. For instance, several studies [3, 4] have incorporated E(3) equivariance into their diffusion model architectures for molecular generation. In the same vein, our diffusion-based model for crystal structure pretraining needs to account for this fundamental constraint, which is discussed in the following.

A.2 Equivariance and Invariance

We investigate the equivariance of geometric models in the group $\mathfrak{G}$, specifically analyzing how the transformation of the input structure affects the output [5, 6, 7]. A function $f : \mathcal{X} \to \mathcal{Y}$ exhibits equivariance if it satisfies $\rho_\mathcal{Y}(\mathfrak{g}) \cdot f(x) = f(\rho_\mathcal{X}(\mathfrak{g}) \cdot x), \forall \mathfrak{g} \in \mathfrak{G}$, where $\rho_\mathcal{X}(\mathfrak{g})$ and $\rho_\mathcal{Y}(\mathfrak{g})$ denote group representations of group element $\mathfrak{g}$ in input and output spaces, respectively. When $\rho_\mathcal{Y}(\mathfrak{g})$ is a trivial representation (*i.e.*, always identity transformation), the function f is invariant to the transformation; otherwise, it is termed equivariant. Hence, invariance can be considered a special case of equivariance. Among those applications in biochemistry, it is a common practice to consider Euclidean transformation (*i.e.*, E(3) Group), including rotation, translation and reflection. Specially owing to the periodic nature of crystalline structures, it is necessary to take periodical translation invariance into account additionally. A thorough discussion of the symmetry considerations is provided as follows:

O(3)-invariance. For any rotation/reflection matrix $\mathbf{O} \in \mathrm{O}(3)$ imposed on the lattice matrix $\vec{\mathbf{L}}$, there is $p(\mathbf{O}\vec{\mathbf{L}}, \mathbf{F}|\mathbf{A}) = p(\vec{\mathbf{L}}, \mathbf{F}|\mathbf{A})$. The distribution remains unchanged no matter how the lattice is transformed under O(3) group.

Periodical translation invariance. Upon translating all atoms by $\mathbf{t} \in \mathbb{R}^{3\times 1}$, the resulting fractional coordinates are expressed as $\{\mathbf{F} + \mathbf{t}\mathbf{1}^\top\}$, with $\{\cdot\}$ representing the fractional part extraction. Then the equation $p(\vec{\mathbf{L}}, \{\mathbf{F} + \mathbf{t}\mathbf{1}^\top\}|\mathbf{A}) = p(\vec{\mathbf{L}}, \mathbf{F}|\mathbf{A})$ follows, illustrating the fact that translating the atom coordinates does not affect the distribution.

For simplicity, we collectively refer to the O(3)-invariance and periodical translation invariance as OP-invariance.

B Theoretical Analysis

In this section, we provide rigorous mathematical proofs and detailed derivations for the key theoretical results presented in the main text.

B.1 Invariance of the learned Node Features

In this part, we provide the details why $\mathbf{H}^{(S)}$ is OP invariant. From Appendix A of DiffCSP [8], we can learn that

$$\boldsymbol{\varphi}_{\texttt{FT}}\left(\{\mathbf{f}_i + \mathbf{t}\} - \{\mathbf{f}_j + \mathbf{t}\}\right) = \boldsymbol{\varphi}_{\texttt{FT}}\left(\mathbf{f}_i - \mathbf{f}_j\right), \tag{16}$$

where, $\mathbf{t} \in \mathbb{R}^{3\times 1}$ is the translation. Then, we prove that the pre-calculated edge feature $\mathbf{e}_{ij}$ exhibits OP-invariance. Given any rotation/reflection matrix $\mathbf{O} \in \mathrm{O}(3)$ imposed on the lattice matrix $\vec{\mathbf{L}}$, we have:

$$\begin{aligned}
\mathbf{e}_{ij}^{\texttt{transformed}} &= \texttt{Normalization}\left((\mathbf{O}\vec{\mathbf{L}})^\top(\mathbf{O}\vec{\mathbf{L}})\ \|\boldsymbol{\varphi}_{\texttt{FT}}\left(\{\mathbf{f}_i + \mathbf{t}\} - \{\mathbf{f}_j + \mathbf{t}\}\right)\right), \\
&= \texttt{Normalization}\left(\vec{\mathbf{L}}^\top(\mathbf{O}^\top\mathbf{O})\vec{\mathbf{L}} \parallel \boldsymbol{\varphi}_{\texttt{FT}}\left(\mathbf{f}_i - \mathbf{f}_j\right)\right) \\
&= \texttt{Normalization}\left(\vec{\mathbf{L}}^\top\vec{\mathbf{L}} \parallel \boldsymbol{\varphi}_{\texttt{FT}}\left(\mathbf{f}_i - \mathbf{f}_j\right)\right) \\
&= \mathbf{e}_{ij}.
\end{aligned} \tag{17}$$

Moreover, the node feature $\mathbf{h}_i^{(0)}$ is OP-invariant, because it is initialized by CGCNN embedding, depending solely on the atom type $\boldsymbol{a}_i$. Therefore, the inputs (i.e. $\boldsymbol{E}, \mathbf{H}^{(0)}$) to our noise prediction model are all OP-invariant. Since the components of our Transformer block, including multi-head attention and feedforward layers, operate exclusively on OP-invariant features, the final output $\mathbf{h}_i^{(S)}$ is also OP-invariant.

B.2 Equivariance of the Noise Output

For fractional coordinates, we compute the score by $\hat{\boldsymbol{\epsilon}}_F(\mathcal{M}_t, t) = \boldsymbol{\varphi}_F(\mathbf{H}_t^{(S)})$. As we have proved that $\mathbf{H}_t^{(S)}$ is OP-invariant in Supplementary Note B.1, it is obvious that the predicted $\hat{\boldsymbol{\epsilon}}_F(\mathcal{M}_t, t)$ also meets OP-invariance. In terms of the lattice noise, we just need to consider O(3) transformation. For $\hat{\boldsymbol{\epsilon}}_L(\mathcal{M}_t, t) = \vec{\mathbf{L}}\boldsymbol{\varphi}_L(\bar{\mathbf{h}}_t)$, if we impose an O(3) transformation $\mathbf{O}$ on the crystal, $\bar{\mathbf{h}}_t$ remains unchanged and the lattice representation becomes $\mathbf{O}\vec{\mathbf{L}}$. We derive the transformed score:

$$\begin{aligned}
\hat{\boldsymbol{\epsilon}}_L^{\texttt{transformed}}(\mathcal{M}_t, t) &= (\mathbf{O}\vec{\mathbf{L}})\boldsymbol{\varphi}_L(\bar{\mathbf{h}}_t) \\
&= \mathbf{O}\left(\vec{\mathbf{L}}\boldsymbol{\varphi}_L(\bar{\mathbf{h}}_t)\right) \\
&= \mathbf{O}\hat{\boldsymbol{\epsilon}}_L(\mathcal{M}_t, t).
\end{aligned} \tag{18}$$

It indicates that the noise $\hat{\boldsymbol{\epsilon}}_L(\mathcal{M}_t, t)$ is also transformed given the transformation on the input crystal. That is, the predicted score for lattice is O(3) equivariant. Since the crystal representation $\bar{\mathbf{h}}_t$ is OP-invariant, the energy output $\boldsymbol{\varphi}_E(\bar{\mathbf{h}}_t)$ maintains OP-invariance as well.

B.3 Derivation of the Intermediate Energy Prediction Loss

From CEP [9], we know that the intermediate energy $\mathcal{E}_t(\mathcal{M}_t) = -\log \mathbb{E}_{q_{0t}(\mathcal{M}_0|\mathcal{M}_t)}[e^{-\beta\mathcal{E}_0(\mathcal{M}_0)}]$, that is $e^{-\mathcal{E}_t(\mathcal{M}_t)} = \mathbb{E}_{q_{0t}(\mathcal{M}_0|\mathcal{M}_t)}[e^{-\beta\mathcal{E}_0(\mathcal{M}_0)}]$. To model $\mathcal{E}_t(\mathcal{M}_t)$ for any $t > 0$, we design a network $f_\phi(\mathcal{M}_t, t)$ to approximate it by the loss function shown in Eq. (12). Here we prove this loss function can lead to the objective.

$$
\begin{aligned}
\mathcal{L}_{\texttt{IEP}}(\phi) &= \mathbb{E}_{q_{0t}(\mathcal{M}_0,\mathcal{M}_t)}\left[\|e^{-f_\phi(\mathcal{M}_t,t)} - e^{-\beta\mathcal{E}_0(\mathcal{M}_0)}\|_2^2\right] \\
&= \mathbb{E}_{q_{0t}(\mathcal{M}_0,\mathcal{M}_t)}\left[e^{-2f_\phi(\mathcal{M}_t,t)} - 2e^{-f_\phi(\mathcal{M}_t,t)-\beta\mathcal{E}_0(\mathcal{M}_0)} + e^{-2\beta\mathcal{E}_0(\mathcal{M}_0)}\right] \\
&= \mathbb{E}_{q_{0t}(\mathcal{M}_0,\mathcal{M}_t)}\left[e^{-2f_\phi(\mathcal{M}_t,t)}\right] - 2\mathbb{E}_{q_{0t}(\mathcal{M}_0,\mathcal{M}_t)}\left[e^{-f_\phi(\mathcal{M}_t,t)-\beta\mathcal{E}_0(\mathcal{M}_0)}\right] + C_1 \\
&= \mathbb{E}_{q_t(\mathcal{M}_t)}\left[e^{-2f_\phi(\mathcal{M}_t,t)}\right] - 2\mathbb{E}_{q_t(\mathcal{M}_t)}\mathbb{E}_{q_{0t}(\mathcal{M}_0|\mathcal{M}_t)}\left[e^{-f_\phi(\mathcal{M}_t,t)-\beta\mathcal{E}_0(\mathcal{M}_0)}\right] + C_1 \\
&= \mathbb{E}_{q_t(\mathcal{M}_t)}\left[e^{-2f_\phi(\mathcal{M}_t,t)} - 2\mathbb{E}_{q_{0t}(\mathcal{M}_0|\mathcal{M}_t)}\left[e^{-f_\phi(\mathcal{M}_t,t)-\beta\mathcal{E}_0(\mathcal{M}_0)}\right]\right] + C_1 \\
&= \mathbb{E}_{q_t(\mathcal{M}_t)}\left[e^{-2f_\phi(\mathcal{M}_t,t)} - 2e^{-f_\phi(\mathcal{M}_t,t)}\mathbb{E}_{q_{0t}(\mathcal{M}_0|\mathcal{M}_t)}\left[e^{-\beta\mathcal{E}_0(\mathcal{M}_0)}\right]\right] + C_1,
\end{aligned}
\tag{19}
$$

where $C_1 = \mathbb{E}_{q_{0t}(\mathcal{M}_0,\mathcal{M}_t)}\left[e^{-2\beta\mathcal{E}_0(\mathcal{M}_0)}\right]$ is a constant independent of the parameter ϕ, and β is the temperature. The optimal minimum of the loss function $\mathcal{L}_{\texttt{IEP}}$ is attained if and only if the condition $e^{-f_\phi(\mathcal{M}_t,t)} = \mathbb{E}_{q_{0t}(\mathcal{M}_0|\mathcal{M}_t)}[e^{-\beta\mathcal{E}_0(\mathcal{M}_0)}]$ is satisfied, indicating that $f_\phi(\mathcal{M}_t, t)$ serves as an approximation of $\mathcal{E}_t(\mathcal{M}_t)$. In the pretraining experiments, we set $\beta = 1$.

C Downstream Datasets Introduction

Matbench [10]. Matbench is a prevailing materials benchmark, tailored for property prediction. It is often used to evaluate various machine learning algorithms. We select JDFT2D [11] to predict Exfoliation Energy, Dielectric [12] to predict Refractive Index and KVRH [13] to predict the average Bulk Moduli. The experimental results are averaged by five folds, which are split by the benchmark in advance.

Materials Project [14]. The Materials Project is a platform that offers access to computed materials data and advanced analysis tools. We follow the datasets used in MEGNet [15], to predict three properties: Shear Modulus, Bulk Modulus and Bandgap. For generation task, MP-20 and MPTS-52 are both sourced from MP. They are curated to contain materials with the atom numbers no more than 20 and 52, respectively.

JARVIS-3D [16]. We use Jarvis-Tools [17] to download the dft3d dataset. The version is aligned with Matformer [18]. We choose properties of Ehull and Bandgap (MBJ), which are two challenging tasks.

Dataset size and the corresponding hyperparameters for each one are introduced in Supplementary Note D.

D Implementation Details

In this section, we present the used hyperparameters of our models and pretrained CSP baselines, and the configurations of finetuning.

D.1 Hyperparameters of DAO

Our pretrained model is a 12-layer Crysformer architecture with a hidden dimension of 384, 8 attention heads, and a SiLU activation function, resulting in a model size of 25M parameters. We adopt the Adam optimizer for training the network and utilize a cosine learning rate scheduler with linear warmup for learning rate adjustment, consistent with current trends in large language models (LLMs) training. Supplementary Table 1 outlines specific hyperparameters during pretraining and finetuning stages, and Supplementary Table 2 lists the resource consumption of pretraining and finetuning (on CSP tasks) process.

Supplementary Table 1. Hyperparameters at pretraining and finetuning stages. CrysDB denotes our Pretraining Dataset. DAO-P (pred.) is intended for property prediction, whereas DAO-P (gen.) is used for structure prediction. "dedup" denotes the deduplicated dataset.

Task	Dataset	#Samples	Learning Rate	Weight Decay	Batch Size × GPUs	Epoch
Pretraining of DAO-P (pred.)	CrysDB	942,884	2e-4	0	1024×2	800
Pretraining of DAO-P (gen.)	CrysDB (dedup)	919,258	2e-4	0	1024×2	800
Pretraining of DAO-G (Stage I)	CrysDB (dedup)	919,258	3e-4	0	1024×3	800
Pretraining of DAO-G (Stage II)	CrysDB (dedup, relaxed)	919,258	1e-4	0	1024×3	500
Property Prediction	JDFT2D	636	8e-5	5e-5	128×1	300
	DIELECTRIC	4,764	5e-5	5e-5	24×2	300
	KVRH	10,987	8e-5	1e-5	128×1	200
	Jarvis_gap	18,171	1e-4	2e-4	256×2	300
	Jarvis_Ehull	55,370	7e-4	1e-4	256×3	500
	Mp_Shear	5,449	1e-4	2e-5	256×2	500
	Mp_Bulk	5,450	3e-4	0	256×2	500
	MP_gap	69,239	2e-5	0	64×3	300
Structure Generation	MP-20	45,231	2e-5	1e-5	400×8	1000
	MPTS-52	40,476	2e-5	1e-5	80×8	1000

Supplementary Table 2. Task and Resource Allocation

Task	Model	GPU Type	GPU Days
Pretrain	DAO-G (Stage I)	A100 (80G)	3.85
	DAO-G (Stage II)	A100 (80G)	3.12
	DAO-P (dedup)	A100 (80G)	3.83
	DAO-P	A100 (80G)	4.52
Finetune	DAO-G (mp-20)	4090 (24G)	0.34
	DAO-G (mpts-52)	4090 (24G)	0.85

D.2 Hyperparameters of Pretrained CSP Baselines

In our experiments, we carefully tuned the hyperparameters for each pretrained CSP baseline on the MP-20 and MPTS-52 datasets. For MatterGen, we employed 2 blocks and used a learning rate of 1×10^{-4} on both datasets; For DiffCSP, the learning rate was set to 5×10^{-5} on MP-20 and 1×10^{-4} on MPTS-52, with a weight decay of 1×10^{-5} applied consistently across both datasets. The larger variant, DiffCSP-large, used a learning rate of 5×10^{-5} on MP-20 and 2×10^{-5} on MPTS-52, also with a weight decay of 1×10^{-5}. For FlowMM, we configured the model with a hidden dimension of 512 and 11 message-passing layers, using a uniform learning rate of 1×10^{-4} on both datasets. Finally, FlowMM-Crysformer was implemented with a hidden dimension of 480 and 5 layers, also trained with a learning rate of 1×10^{-4} on MP-20 and MPTS-52. All unspecified parameters were kept at their default values.

D.3 Configurations of Finetuning

For crystal property prediction downstream tasks, a two-layer MLP (i.e., prediction head) is integrated with the pretrained model, allowing for simultaneous finetuning of the entire model. Separate prediction heads are used for each dataset and when training we normalize the property

Supplementary Table 3. Experimental results (averaged on three runs) for CSP task across different DAO-G configurations. Here, "Stage I" denotes first-stage-only pretraining, "stable" represents pretraining using the 306,830 stable crystals, "only relax" means using the relaxed data only, and "w/ guidance" indicates using energy-guided sampling.

	# of samples	MP-20		MPTS-52	
		Match Rate (%) ↑	RMSE ↓	Match Rate (%) ↑	RMSE ↓
DAO-G (Stage I, stable)	1	65.63 ± 0.07	0.0434 ± 0.0003	31.17 ± 0.11	0.0790 ± 0.0015
DAO-G (Stage I)	1	65.60 ± 0.22	0.0411 ± 0.0005	32.52 ± 0.18	0.0731 ± 0.0008
DAO-G (Stages I+II)	1	**65.97 ± 0.22**	0.0401 ± 0.0013	32.59 ± 0.10	0.0695 ± 0.0013
DAO-G (Stages I+II, w/ guidance)	1	65.65 ± 0.18	0.0406 ± 0.0014	**32.78 ± 0.06**	0.0688 ± 0.0023
DAO-G (Stage I, only relax)	1	58.37 ± 0.25	0.0605 ± 0.0019	23.78 ± 0.16	0.0964 ± 0.0004
DAO-G (Stage I+II (stable+relax))	1	65.29 ± 0.14	**0.0387 ± 0.0002**	32.16 ± 0.03	**0.0685 ± 0.0011**
DAO-P (gen.)	1	63.01 ± 0.02	0.0480 ± 0.0007	29.73 ± 0.20	0.0819 ± 0.0007

labels to follow a standard normal distribution for better numerical stability.

While for CSP tasks, we finetune the model using the same loss functions as the pretraining. After finetuning, we generate structures with the test set and evaluate them against the ground truth by calculating the MR and RMSE.

E Extended Experimental Results

E.1 Deeper Analysis of Structure Generation

In this section, we present further results on the CSP task, including ablation studies of DAO-G and its 20-shot sampling performance.

E.1.1 Ablations on Two-Stage Pretraining

The finetuning results for various DAO configurations on the MP-20 and MPTS-52 datasets are presented in Supplementary Table 3. It can be observed that:

- Stage I vs. Stage I (stable). We compare two settings for Stage I pretraining: (i) using the full deduplicated dataset (including unstable structures) and (ii) using only the stable subset. The results show that, except for the Match Rate metric on MP-20 where the two settings are comparable, the full-dataset model consistently outperforms the stable-only model across other benchmarks. This confirms that incorporating unstable structures is indeed reasonable.
- DAO-G (Stage I) vs. DAO-P (gen.). Since DAO-P is trained with both structure generation and energy prediction losses, in principle it is capable of crystal structure generation. However, DAO-P is optimized under a composite objective—balancing both energy regression and denoising for structure generation—within the same model capacity as DAO-G. This trade-off inevitably weakens its ability in structure generation compared to a dedicated model. To verify this, we directly evaluated DAO-P on the CSP task. The results show that DAO-P underperforms DAO-G (Stage I) across all metrics. This confirms that although DAO-P can generate structures, its generative performance is compromised, which motivates training DAO-G as a separate, specialized generator.
- Stage I+II vs. Stage I+II (stable+relax). To verify whether the unstable structures still valuable for training, we remove unstable structures entirely, i.e., only using stable + relaxed

data in Stage II pretraining. The results indicate a trade-off between Match Rate and RMSE: performance slightly declines in terms of Match Rate while improving in terms of RMSE. For instance, compared to using the full dataset, the Match Rate decreases from 65.97% to 65.29% on MP-20 and from 32.59% to 32.16% on MPTS-52, while the RMSE shows a corresponding improvement. This suggests that incorporating unstable (unrelaxed) structures contributes to the generation of more reasonable structures (as reflected in the higher Match Rate), even though it leads to a marginal degradation in RMSE.

- Stage I vs. Stage I (only relax). We conducted an additional experiment where we used an untrained DAO-G model and pretrained it solely on the augmented Stage II dataset. This was done to verify whether the improvements come mainly from DAO-P imputing structures during relaxation. While this approach achieved reasonable results, the performance was consistently lower than that of both our Stage I–pretrained and Stage II-pretrained DAO-G. For example, compared to DAO-G (Stage I), the Match Rate dropped from 65.60% to 58.37% on MP-20, and from 32.52% to 23.78% on MPTS-52. These results indicate that our two-stage pretraining strategy, as well as retaining non-relaxed data in Stage I, is crucial for our framework's superior performance.

E.1.2 Ablations on Energy Relaxation Threshold

In Stage II pretraining, we selected only structures with Ehull in the range (0.08,0.5] eV/atom for relaxation. The rationale is as follows:

- Structures with Ehull>0.5 eV/atom exhibit poor thermodynamic stability and are far from the ground-state configuration. Relaxing such structures often requires a larger optimization step size, which may adversely affect the optimization of lower-energy structures. Considering both computational efficiency and stability, we excluded structures with Ehull>0.5 eV/atom from relaxation.

- The choice of the 0.5 threshold was initially empirical, as it lies near the midpoint of the Ehull distribution in our pretraining dataset ([0, 1.0] eV/atom).

To further validate this choice, we conducted an ablation study by varying the relaxation threshold among 0.0 (no relaxation), 0.3, 0.5, 0.7, and 1.0 (relax all structures), while keeping the same L-BFGS parameters described in Section 4.5. The results (Supplementary Fig. 1) show that:

- Compared with no relaxation (threshold = 0.0), the threshold of 0.5 yields a higher Match Rate, suggesting that incorporating more relaxed structures in pretraining helps the model generate more physically plausible structures. RMSE is slightly higher, which we attribute to the model's exposure to more successfully matched structures (as Match Rate is higher), increasing the diversity of atomic coordinates after relaxation.

- For all thresholds greater than zero, the Match Rate increases on both datasets as the threshold is raised. Although a threshold of 1.0 yields the highest Match Rate, it also results in a relatively high RMSE on MPTS-52. Considering the trade-offs between all four metrics across both datasets, we found a threshold of 0.5 to offer the most balanced and reasonable performance.

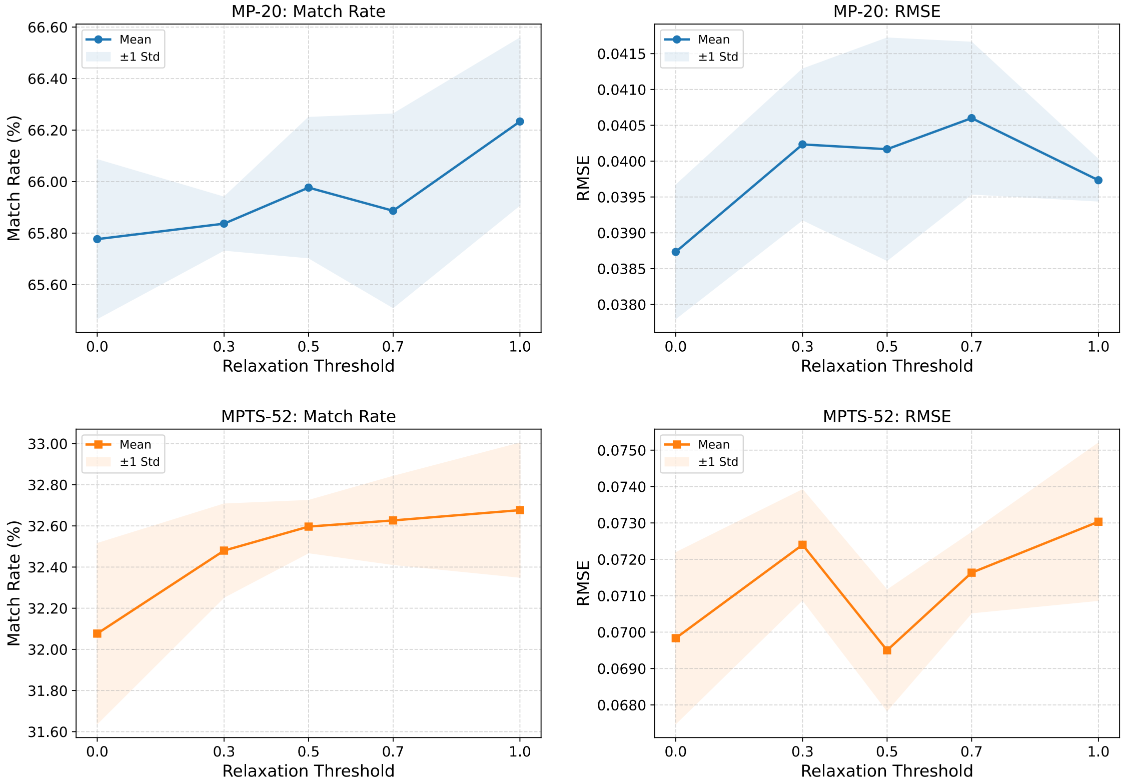

Supplementary Figure 1. The influence of relaxation thresholds. The standard deviations are calculated across three runs.

Overall, a threshold of 0.5 strikes the best balance between high Match Rate, low RMSE, and reasonable relaxation cost, as it avoids excessive relaxation of very unstable structures while still improving performance.

Moreover, we have performed a detailed analysis of the structural changes during dataset relaxation. Specifically, we relaxed 429,731 structures with Ehull in the range (0.08, 0.5] eV/atom. After relaxation, we reapplied the same deduplication procedure described in Section 4.2. We found that only 8 relaxed structures matched any entries in the downstream test sets (which contain 17,142 structures in total, i.e., 9,046 in MP-20 and 8,096 in MPTS-52). This proportion is extremely small, suggesting that the impact of dataset relaxation on potential data leakage is negligible.

E.1.3 20-shot Results

We also perform 20-shot sampling for the CSP evaluation and compare DAO-G against several non-pretrained baseline methods. As shown in Supplementary Table 4, DAO-G achieves state-of-the-art performance across all evaluation metrics on both the MP-20 and MPTS-52 datasets. These results demonstrate the effectiveness and superiority of DAO-G under a multiple-sampling setting.

E.2 Accurate Crystal Property Prediction via the Finetuned DAO-P

In the main context, DAO-P has functioned as an energy predictor, assisting DAO-G through dataset relaxation and sampling guidance. We now explore whether the pretraining of DAO-P on energy prediction provides a robust foundation for predicting a broader spectrum of material properties. Specifically, we first pretrain DAO-P on the full version of CrysDB and then finetune it on eight representative datasets selected from three widely-used benchmarks: Matbench [10], JARVIS-3D [16]

Supplementary Table 4. A Comparison of 20-shot sampling Results.

	MP-20		MPTS-52	
	**Match Rate (%) ↑**	**RMSE ↓**	**Match Rate (%) ↑**	**RMSE ↓**
CDVAE	66.95	0.1026	20.79	0.2085
DiffCSP	77.93	0.0492	34.02	0.1749
FlowMM	75.81	0.0479	34.05	0.1813
CrystalFlow	78.34	0.0577	40.37	0.1576
DAO-G (w/o pretrain)	76.49	0.0600	35.44	0.1447
DAO-G	**82.68**	**0.0279**	**46.78**	**0.0795**

and MP [14]. To ensure consistency and comparability, we adopt the experimental settings described in Matformer [18] for JARVIS-3D and MP, and utilize the default settings for the Matbench datasets. For comparison, we choose two categories of SOTA methods on these benchmarks: models without pretraining [15, 18, 19, 20, 21, 22], and models with pretraining [23, 24, 25]. We quantify the accuracy using the MAE metric.

The results are presented in Supplementary Fig. 2. Notably, DAO-P outperforms previous approaches on half of the evaluated datasets and achieves competitive results on the remaining datasets, with the exception of MP_gap. Across these datasets, DAO-P consistently ranks among the top three performers, demonstrating its robustness and generalization capability. Particularly striking is the significant performance improvement on Jarvis_Ehull, where DAO-P achieves a 16.3% increase over the second-best method. Furthermore, the effectiveness of DAO-P in few-shot learning scenarios is demonstrated by its outstanding performance on the JDFT2D dataset, which contains only 636 instances. In conclusion, DAO-P is not limited to energy prediction; it can be adapted to predict a wide range of material properties with appropriate finetuning datasets. Moreover, the principle of energy guidance can be extended to other properties. For instance, DAO-P could be utilized to bias the generation process toward structures with enhanced Shear Modulus.

In addition, we further investigated the impact of pretraining by applying our same pretraining dataset to two of the train-from-scratch baselines: SchNet [19] and MEGNet [15]. It should be noted that the original results of these two models were reproduced from MMPT [25]. However, since MMPT is not open-sourced, we are unable to directly access its code for pretraining. As stated in the MMPT appendix, the results on the three Matbench datasets were obtained from its website, while other results were reproduced from Matformer. Although Matformer provides model parameters, it does not release the training code for these two models. As a result, different hyperparameters were used across datasets, and the lack of accessible code posed challenges for reproduction. The primary aim of this experiment is to validate the performance improvement attributable to pretraining. Therefore, it is paramount to maintain identical code implementations both before and after pretraining. For this, we used public versions from PyG and DeepChem to ensure differences are not due to code or configuration. We also scaled their parameters (SchNet: 25.8M, MEGNet: 25.2M) to match our DAO-P model (25.2M) closely.

As a result, this part of the evaluation will differ from Supplementary Table 5, and we make this clarification to avoid confusion. The updated results are shown in Supplementary Table 6 and the corresponding training hyperparameters are presented in Supplementary Table 7. Across

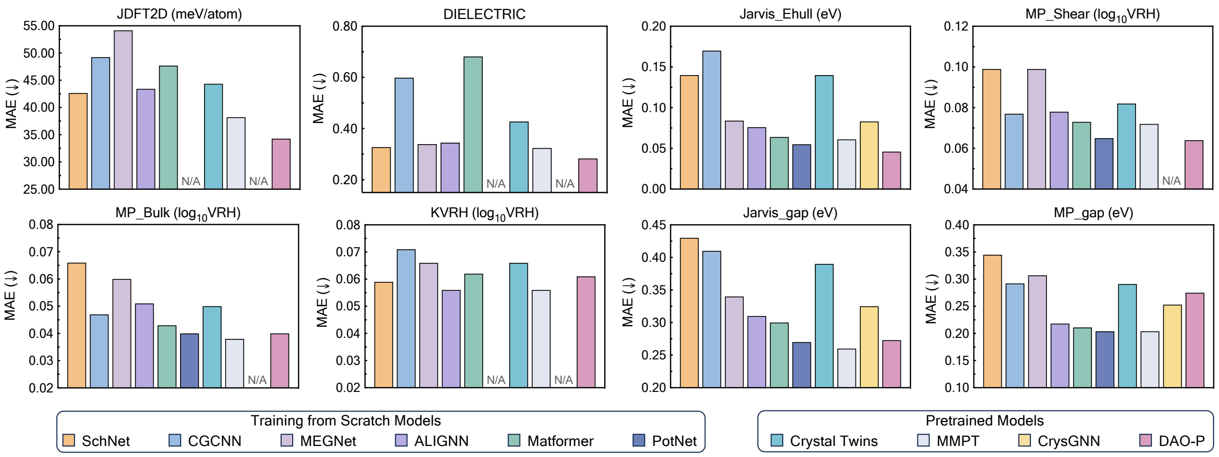

Supplementary Figure 2. The performance of DAO-P for crystal property prediction is evaluated on eight datasets. The compared baselines include models both with and without pretraining. The results for PotNet [22] and CrysGNN [23] are obtained from their original publications, while the results for the remaining baseline models are sourced from the MMPT paper [25]. For baselines where the corresponding experiments were not conducted in the original paper, the results are denoted as N/A. The raw results are recorded in Supplementary Table 5.

Supplementary Table 5. Experimental results (MAE ↓) of crystal property prediction on eight datasets.

	**MatBench**			**JARVIS-3D**		**MP**		
	JDFT2D (meV/atom)	DIELECTRIC	KVRH (log_{10} VRH)	Jarvis_gap (eV)	Jarvis_Ehull (eV)	Mp_Shear (log_{10} VRH)	Mp_Bulk (log_{10} VRH)	MP_gap (eV)
SchNet	42.663	0.327	<u>0.059</u>	0.430	0.140	0.099	0.066	0.345
CGCNN	49.244	0.598	0.071	0.410	0.170	0.077	0.047	0.292
MEGNet	54.171	0.339	0.066	0.340	0.084	0.099	0.060	0.307
ALIGNN	43.424	0.344	**0.056**	0.310	0.076	0.078	0.051	0.218
Matformer	47.696	0.681	0.062	0.300	0.064	0.073	0.043	<u>0.211</u>
PotNet	-	-	-	<u>0.270</u>	<u>0.055</u>	<u>0.065</u>	0.040	**0.204**
Crystal Twins	44.353	0.427	0.066	0.390	0.140	0.082	0.050	0.291
MMPT	<u>38.213</u>	<u>0.324</u>	**0.056**	**0.260**	0.061	0.072	**0.038**	**0.204**
CrysGNN	-	-	-	0.325	0.083	-	-	0.253
DAO-P	**34.280**	**0.283**	0.061	0.273	**0.046**	**0.064**	<u>0.040</u>	0.275

eight downstream property prediction datasets, both SchNet and MEGNet generally exhibit lower MAE after pretraining (with the exception of SchNet on MP_Bulk, which shows a slight increase). However, even after pretraining on the same dataset, both models still underperform DAO-P, which highlights the strong effectiveness of our DAO-P model.

E.3 Additional Results on Superconductor Validation

In this section, we provide further experimental details regarding the validation on superconductors, including implementation specifics (e.g., hyperparameter settings) and corresponding DFT results.

Supplementary Table 6. The downstream property prediction results (MAE↓) of pretrained baselines.

	MatBench			JARVIS-3D		MP		
	JDFT2D (meV/atom)	DIELECTRIC	KVRH ($\log_{10}$ VRH)	Jarvis_gap (eV)	Jarvis_Ehull (eV)	Mp_Shear ($\log_{10}$ VRH)	Mp_Bulk ($\log_{10}$ VRH)	MP_gap (eV)
SchNet	65.912	0.417	0.104	0.635	0.205	0.106	0.069	0.412
SchNet (Pretrain)	63.503	0.402	0.095	0.444	0.177	0.091	0.072	0.379
MEGNet	67.143	0.360	0.081	0.429	0.159	0.105	0.073	0.351
MEGNet (Pretrain)	54.713	0.349	0.073	0.403	0.144	0.101	0.068	0.338
Our	**34.280**	**0.283**	**0.061**	**0.273**	**0.046**	**0.064**	**0.040**	**0.275**

Supplementary Table 7. The hyperparameters of SchNet and MEGNet.

	MatBench			JARVIS-3D		MP		
	JDFT2D (meV/atom)	DIELECTRIC	KVRH ($\log_{10}$ VRH)	Jarvis_gap (eV)	Jarvis_Ehull (eV)	Mp_Shear ($\log_{10}$ VRH)	Mp_Bulk ($\log_{10}$ VRH)	MP_gap (eV)
SchNet	lr=0.0001 decay=0.0001	lr=0.0005 decay=0.0005	lr=0.001 decay=0.0001	lr=0.001 decay=0.001	lr=0.0005 decay=0.0001	lr=0.001 decay=0.0001	lr=0.0003 decay=0.0	lr=0.0005 decay=0.0001
SchNet (Pretrain)	lr=0.0005 decay=0.00005	lr=0.0001 decay=0.0001	lr=0.0001 decay=0.001	lr=0.0001 decay=0.0002	lr=0.0005 decay=0.0001	lr=0.0001 decay=0.00005	lr=0.001 decay=0.00005	lr=0.001 decay=0.0001
MEGNet	lr=0.0001 decay=0.00005	lr=0.001 decay=0.0001	lr=0.001 decay=0.0001	lr=0.001 decay=0.0001	lr=0.0005 decay=0.0001	lr=0.0005 decay=0.0001	lr=0.001 decay=0.0001	lr=0.0005 decay=0.0005
MEGNet (Pretrain)	lr=0.001 decay=0.0001	lr=0.001 decay=0.0001	lr=0.0001 decay=0.00005	lr=0.001 decay=0.001	lr=0.0005 decay=0.0001	lr=0.0005 decay=0.0001	lr=0.0005 decay=0.0001	lr=0.001 decay=0.0005

Supplementary Table 8. The best configuration of each T_c predictor and the corresponding MAE (logK). "aug." denotes the model is augmented by generated structures.

Model	LR	Decay	Fold 1	Fold 2	Fold 3	Fold 4	Fold 5	Average
DAO-P (w/o aug.)	2e-4	5e-5	0.838 ± 0.016	0.755 ± 0.021	0.694 ± 0.003	0.763 ± 0.017	0.758 ± 0.012	0.761 ± 0.011
DAO-P (aug.)	4e-4	5e-5	**0.823 ± 0.009**	**0.695 ± 0.016**	**0.636 ± 0.006**	**0.718 ± 0.021**	**0.699 ± 0.007**	**0.714 ± 0.004**

Supplementary Table 9. T_c prediction results on three real-world superconductors.

Formula	Experimental T_c (K)	Predicted T_c (K)	Absolute Error (K)
Cr_6Os_2 [26]	3.99	1.97	2.02
$Zr_{16}Pd_8O_4$ [27]	2.73	2.99	0.26
$Zr_{16}Rh_8O_4$ [27]	3.73	3.77	0.04

E.3.1 Joint Superconducting Property Prediction and Structure Generation

In Section 4.8, we train DAO-P with and without augmented data, respectively. For each setting, we conduct a grid search over learning rates (2e-4, 3e-4, and 4e-4) and weight decay values (1e-5, 2e-5, 3e-5, and 5e-5), and then select the respective best model for comparison in Supplementary Table 8. As expected, the augmentation with generated structures significantly improves the performance of DAO-P, resulting in the best performance on all five folds.

Additionally, we extend our experiments to three real-world superconductors, utilizing the

Supplementary Table 10. The configurations of DFT calculation.

system				electrons	cell		ions
ecutwfc	occupations	smearing	degauss	conv_thr	press_conv_thr	cell_dynamics	ion_maxstep
40.0	smearing	gaussian	0.01d0	1.0d-6	0.5	bfgs	50 (default)

Supplementary Table 11. The results of 20 runs of DFT calculations. "N/A" denotes the failed match.

Run-id	1	2	3	4	5	6	7	8	9	10
RMSE	0.0414	0.2716	N/A	0.2961	0.0418	0.0410	0.2062	0.0414	N/A	0.0412
Run-id	11	12	13	14	15	16	17	18	19	20
RMSE	0.3375	N/A	N/A	0.0416	N/A	**0.0410**	0.0416	0.3437	0.0412	0.1377

Supplementary Table 12. The results of 20 runs of DAO-G generation.

Run-id	1	2	3	4	5	6	7	8	9	10
RMSE	0.0017	0.0018	0.0015	0.0017	0.0016	0.0017	0.0016	0.0016	0.0018	0.0015
Run-id	11	12	13	14	15	16	17	18	19	20
RMSE	0.0015	0.0015	0.0019	0.0016	0.0016	**0.0012**	0.0020	0.0016	0.0014	0.0016

Supplementary Table 13. A comparison between DFT and our DAO-G on the generation of Cr_6Os_2 [26].

Method	Initialized Structure	RMSE	Iterations	Running Time
DFT [28]	perturbation on the experiment structure<br>(10% noise for abc, 10° noise for $\alpha\beta\gamma$, 0.1 noise for $\mathbf{F}$)	0.0410	38 scf-steps<br>+ 37 bfgs-steps	138.74m
DAO-G	absolute noises sampled from $\mathcal{U}(0, 1)$ and $\mathcal{N}(\mathbf{0}, \mathbf{I})$	0.0012	1000	1.50m

previously finetuned DAO-G and DAO-P (aug.) models. We first employ the ensemble DAO-P models to predict T_c for them and the results on Supplementary Table 9 indicate the predicted T_c values are consistent with the experimentally determined values. Then DAO-G is used to generate structures for the three superconductors, given only their formulas, with 20-shot sampling. Further, we select the sample with the lowest RMSE from the generated 20 samples for each superconductor, and visualize them in the second row of Fig. 4d. Impressively, all three generated structures show excellent agreement with the ground truth structures. These findings suggest that models finetuned on SuperCon exhibits strong generalization to newly discovered superconductors.

E.3.2 DFT Results for Three Real-World Superconductors

We additionally employ the Quantum-Espresso software (QE [28]), a prevailing DFT tool, to calculate the relaxed structures for the three real-world superconductors. The configurations of QE calculation are presented in Supplementary Table 10. To ensure a fair comparison, the input structures are randomly initialized, consistent with our diffusion-based generation process. However, all three generation attempts are unsuccessful. We hypothesize that this failure stems from the structural complexity of these superconductors. Therefore, to reduce the computational burden, we perturb the ground-truth structures slightly before employing them as starting points for QE

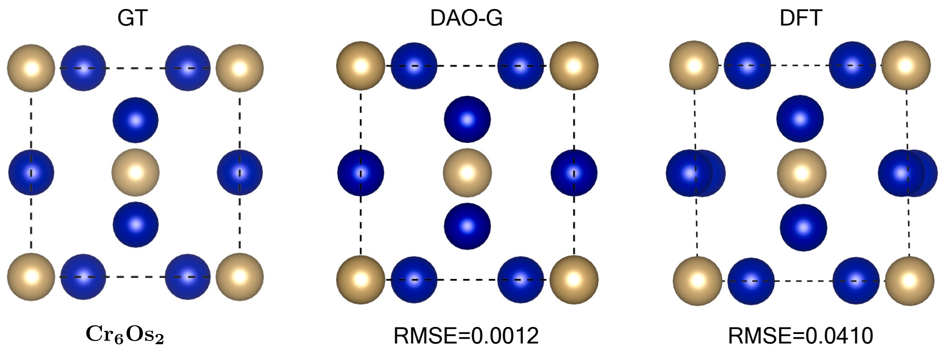

Supplementary Figure 3. An illustration of the structure relaxed by DFT and the structure generated by DAO-G, for the superconductor Cr_6Os_2 [26]. GT is the ground truth structure.

calculations. Specifically, we apply 10% lattice lengths perturbation, 10-degree angles perturbation, and 0.1 fractional coordinates deviation. For Cr_6Os_2 [26], across 20 runs, QE calculations results in five failed matches, whereas DAO-G achieves success in all runs. As shown in Supplementary Tables 11 and 12, the best result generated by DAO-G is 0.0012, which is significantly superior to the best QE calculation result of 0.0410. The visualizations of the corresponding best results are depicted in Supplementary Fig. 3. Clear discrepancies exist between the relaxed structure and the ground truth, while the structure generated by DAO-G shows well alignment. Interestingly, although DAO-G has seen unstable Cr_6Os_2 structures during pretraining (no data leakage), it does not bias toward reproducing these unstable configurations at generation time. Instead, DAO-G generates the stable superconducting structure, with a DFT-calculated Ehull of 0.02918, closely matching the experimental value (0.02916). This behavior indicates that DAO-G is not merely memorizing training examples, but has learned a meaningful distribution over stable crystal structures conditioned on composition, even when unstable polymorphs of the same formula are present in the training data.

Regarding efficiency, DAO-G is approximately 92 times faster than DFT calculations. DAO-G generates a structure in 1.5 minutes, while QE requires approximately 2 hours and 18 minutes. The comparison is presented in Supplementary Table 13. For the two more complex superconductors ($Zr_{16}Pd_8O_4$ [27] and $Zr_{16}Rh_8O_4$ [27]), even slight perturbations similar to those applied to Cr_6Os_2 results in computational exceptions. Subsequently, we further decreased the perturbations to 5% for lattice parameters, 3 degrees for angles, and a standard deviation of 0.05 for fractional coordinates. However, each iteration still requires over 8 hours of computation time, and the Self-Consistent Field (SCF) process fails to converge within 8 days, exceeding practical time constraints.

F Visualization

F.1 Visualization of Generated Polymorphs

In Supplementary Fig. 4, we also provide visualizations for cases involving 2- and 3-polymorphs. The results demonstrate that our model successfully generates diverse structural configurations for each case, highlighting its strong capability in modeling complex polymorphic distributions.

F.2 Visualization of the Diffusion Process

To better understand how the structures evolve during the generation process, we select several examples from MP-20 dataset and visualize the perturbed structures at different timesteps in

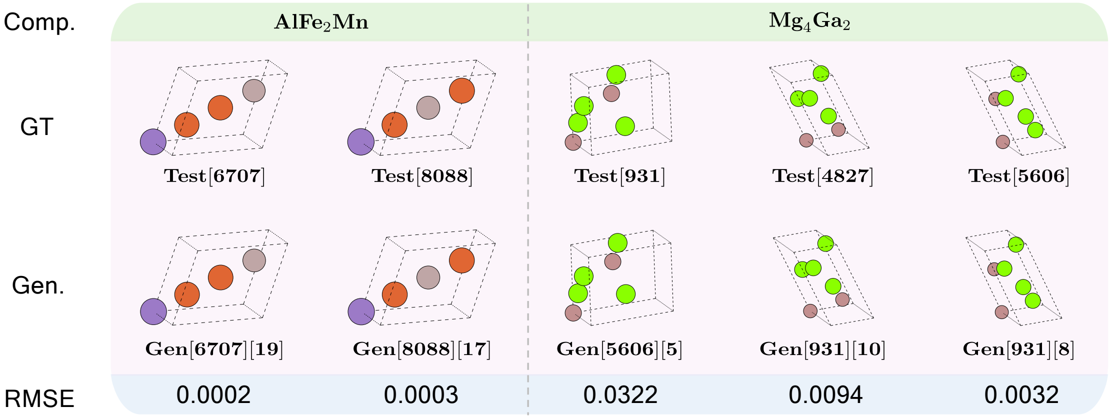

Supplementary Figure 4. The visualization of the polymorphs (with 2 and 3 conformations) generated by DAO-G, with the corresponding ground-truth structures. Comp. = Composition, GT = Ground Truth, Gen. = Generation.

Supplementary Fig. 5.

G Comparison with DiffCSP and MatterGen

Our method, DAO, shares certain similarities with both DiffCSP [8] and MatterGen [29]. To further clarify our contributions, this section elaborates on the key distinctions between DAO and each of these approaches.

G.1 Comparative Analysis with DiffCSP

Although our method is closely linked to DiffCSP, important differences exist. Below we discuss both the commonalities and distinctions.

- **Relevance.** Both our DAO-G and DiffCSP target the CSP task and employ a joint diffusion process to generate both lattice and fractional coordinates.
- **Differences.** Despite these shared ideas, our framework substantially differs from DiffCSP in three aspects:

  1. **Training paradigm.** DiffCSP is trained entirely from scratch, while our framework follows a pretrain–finetune paradigm. We conduct a two-stage pretraining on a large-scale crystal dataset (CrysDB, 910k structures with both structures and energies) and then finetune on the DiffCSP benchmark datasets, leading to stronger performance.
  2. **Backbone architecture.** DiffCSP adopts a GNN-based backbone (similar to EGNN [30]), whereas our framework introduces a Transformer-based backbone (Crysformer), which provides stronger expressivity and generalization ability, especially under large-scale pretraining.
  3. **Energy modeling.** DiffCSP briefly discusses energy optimization (Appendix G.3), where the energy at intermediate steps is supervised directly using the ground-truth energy at $t = 0$, i.e., $\mathcal{L}_{\text{DiffCSP}} = \|f_\phi(\mathcal{M}_t, t) - \mathcal{E}_0(\mathcal{M}_0)\|_2^2$. This approach has known limitations (presented in Section 4.5), such as inaccurate prediction or numerical

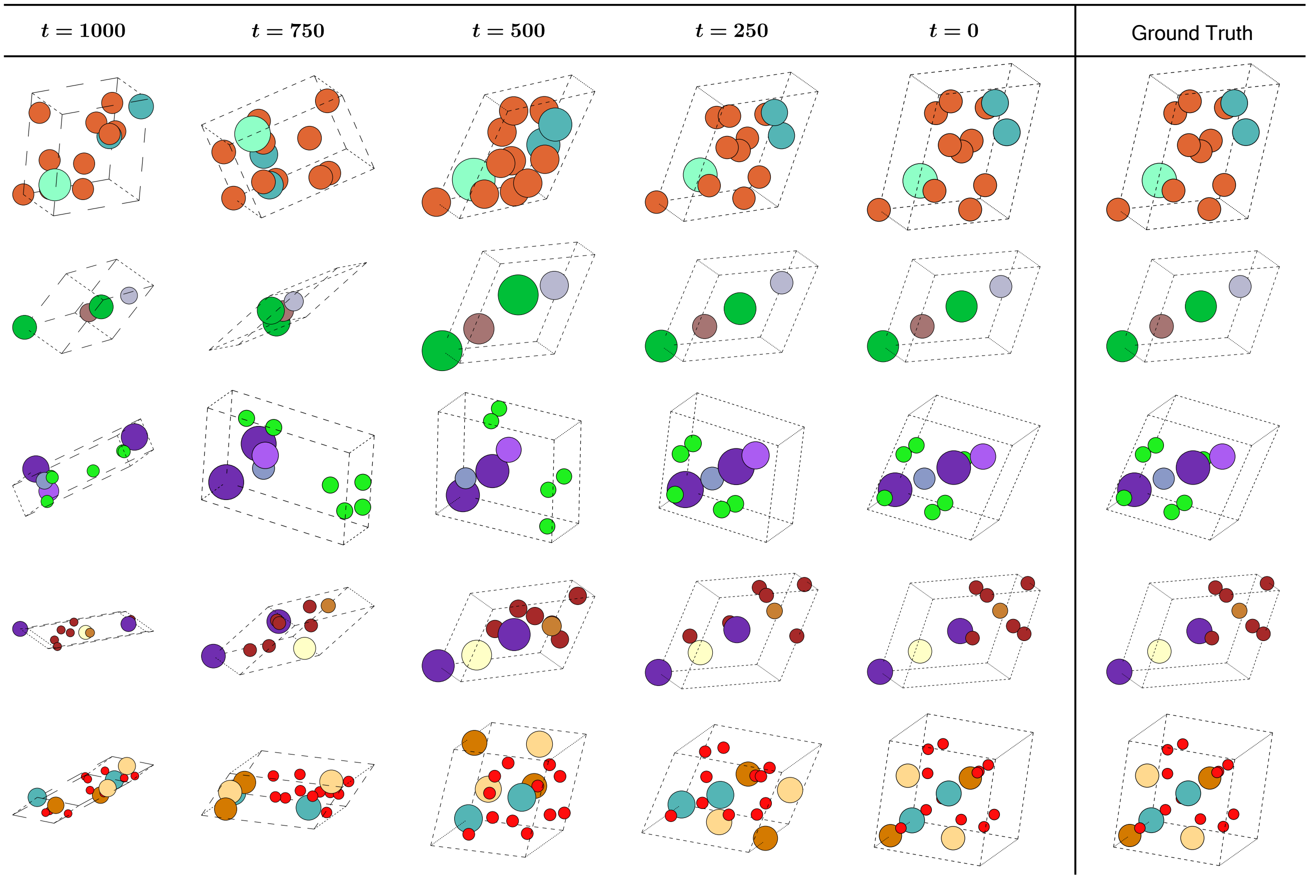

Supplementary Figure 5. Visualizations of the generated structures by DAO-G throughout the diffusion process. We show representative structures at timesteps 1000, 750, 500, 250, and 0. The structures at timestep 0 represent the final generated samples, which are well-aligned with the corresponding ground truth structures. To enhance visual clarity and facilitate comparison, a common atom within each group (represented by a row) has been translated to the origin.

instability. In contrast, we propose a new exponential-based energy loss: $\mathcal{L}_{\text{DAO}} = \|e^{-f_\phi(\mathcal{M}_t,t)} - e^{-\mathcal{E}_0(\mathcal{M}_0)}\|_2^2$, which we have justified theoretically as the optimal formulation (see Supplementary Note B.3).

G.2 Comparative Analysis with MatterGen

MatterGen is a well-known work in crystal generation and is relevant to our study. However, we would like to emphasize that MatterGen's pretraining primarily targets *de novo* generation (DNG), where both composition and structure are generated jointly. In contrast, DAO is designed for Crystal Structure Prediction (CSP), where the composition is given and the goal is to generate the corresponding structure. While MatterGen can be adapted for CSP, this is only a minor aspect explored in their work and is not the main focus.

On the other hand, Our DAO-P model and the classifier-free guidance (CFG) approach in MatterGen both provide guidance but differ fundamentally in their nature and purpose:

1. **Model type:** DAO-P is an explicit energy predictor, a neural network trained to estimate the energy of intermediate structures in the reverse process of diffusion. In contrast, CFG,

despite its name including classifier, is not an explicit predictor; it is a training strategy that does not require a separate model to make predictions.

2. **Underlying principle:** The difference in working mechanisms is closely tied to the tasks each method addresses. Our work focuses on the Crystal Structure Prediction (CSP) task, where the goal is to generate stable crystal structures with low energy. As a result, DAO-P is designed to predict the energy and computes its gradient with respect to the structure, using this information to guide generation toward lower-energy configurations. In MatterGen, however, the goal is conditional crystal generation (e.g., conditioned on chemistry and symmetry), and CFG embeds the provided conditions into the generative model to guide the output toward satisfying those conditions.

3. **Implementation details:** DAO-P takes a crystal structure as input and outputs its predicted energy. CFG, on the other hand, encodes the condition via a lightweight adapter module, concatenates this encoding with the structure's representation, and feeds the combined input into the generative model. During training, conditions are randomly dropped with a certain probability, but there is still only one generative model plus the adapter module—no separate predictor is involved.

Supplementary References

1. Jonathan Ho, Ajay Jain, and Pieter Abbeel. Denoising diffusion probabilistic models. *Advances in neural information processing systems*, 33:6840–6851, 2020.

2. Yang Song, Conor Durkan, Iain Murray, and Stefano Ermon. Maximum likelihood training of score-based diffusion models. *Advances in neural information processing systems*, 34:1415–1428, 2021.

3. Emiel Hoogeboom, Vıctor Garcia Satorras, Clément Vignac, and Max Welling. Equivariant diffusion for molecule generation in 3d. In *International conference on machine learning*, pages 8867–8887. PMLR, 2022.

4. Lei Huang, Hengtong Zhang, Tingyang Xu, and Ka-Chun Wong. Mdm: Molecular diffusion model for 3d molecule generation. In *Proceedings of the AAAI Conference on Artificial Intelligence*, volume 37, pages 5105–5112, 2023.

5. Jiacheng Cen, Anyi Li, Ning Lin, Yuxiang Ren, Zihe Wang, and Wenbing Huang. Are high-degree representations really unnecessary in equivariant graph neural networks? *Advances in Neural Information Processing Systems*, 37:26238–26266, 2024.

6. Vıctor Garcia Satorras, Emiel Hoogeboom, and Max Welling. E (n) equivariant graph neural networks. In *International conference on machine learning*, pages 9323–9332. PMLR, 2021.

7. Jiacheng Cen, Anyi Li, Ning Lin, Tingyang Xu, Yu Rong, Deli Zhao, Zihe Wang, and Wenbing Huang. Universally invariant learning in equivariant gnns. *Advances in Neural Information Processing Systems*, 2025.

8. Rui Jiao, Wenbing Huang, Peijia Lin, Jiaqi Han, Pin Chen, Yutong Lu, and Yang Liu. Crystal structure prediction by joint equivariant diffusion. *Advances in Neural Information Processing Systems*, 36, 2024.

9. Cheng Lu, Huayu Chen, Jianfei Chen, Hang Su, Chongxuan Li, and Jun Zhu. Contrastive energy prediction for exact energy-guided diffusion sampling in offline reinforcement learning. In *International Conference on Machine Learning*, pages 22825–22855. PMLR, 2023.

10. Alexander Dunn, Qi Wang, Alex Ganose, Daniel Dopp, and Anubhav Jain. Benchmarking materials property prediction methods: the matbench test set and automatminer reference algorithm. *npj Computational Materials*, 6(1):138, 2020.

11. Kamal Choudhary, Irina Kalish, Ryan Beams, and Francesca Tavazza. High-throughput identification and characterization of two-dimensional materials using density functional theory. *Scientific reports*, 7(1):5179, 2017.

12. Ioannis Petousis, David Mrdjenovich, Eric Ballouz, Miao Liu, Donald Winston, Wei Chen, Tanja Graf, Thomas D Schladt, Kristin A Persson, and Fritz B Prinz. High-throughput screening of inorganic compounds for the discovery of novel dielectric and optical materials. *Scientific data*, 4(1), 2017.

13. Maarten De Jong, Wei Chen, Thomas Angsten, Anubhav Jain, Randy Notestine, Anthony Gamst, Marcel Sluiter, Chaitanya Krishna Ande, Sybrand Van Der Zwaag, Jose J Plata, et al. Charting the complete elastic properties of inorganic crystalline compounds. *Scientific data*, 2(1):1–13, 2015.

14. A Jain, SP Ong, G Hautier, W Chen, WD Richards, S Dacek, S Cholia, D Gunter, D Skinner, G Ceder, et al. The materials project: a materials genome approach to accelerating materials innovation. apl mater 1: 011002, 2013.

15. Chi Chen, Weike Ye, Yunxing Zuo, Chen Zheng, and Shyue Ping Ong. Graph networks as a universal machine learning framework for molecules and crystals. *Chemistry of Materials*, 31(9):3564–3572, 2019.

16. Kamal Choudhary, Kevin F Garrity, Andrew CE Reid, Brian DeCost, Adam J Biacchi, Angela R Hight Walker, Zachary Trautt, Jason Hattrick-Simpers, A Gilad Kusne, Andrea Centrone, et al. The joint automated repository for various integrated simulations (jarvis) for data-driven materials design. *npj computational materials*, 6(1):173, 2020.

17. Kamal Choudhary, Daniel Wines, Kangming Li, Kevin F Garrity, Vishu Gupta, Aldo H Romero, Jaron T Krogel, Kayahan Saritas, Addis Fuhr, Panchapakesan Ganesh, et al. Jarvis-leaderboard: a large scale benchmark of materials design methods. *npj Computational Materials*, 10(1):93, 2024.

18. Keqiang Yan, Yi Liu, Yuchao Lin, and Shuiwang Ji. Periodic graph transformers for crystal material property prediction. *Advances in Neural Information Processing Systems*, 35:15066–15080, 2022.

19. Kristof Schütt, Pieter-Jan Kindermans, Huziel Enoc Sauceda Felix, Stefan Chmiela, Alexandre Tkatchenko, and Klaus-Robert Müller. Schnet: A continuous-filter convolutional neural network for modeling quantum interactions. *Advances in neural information processing systems*, 30, 2017.

20. Tian Xie and Jeffrey C Grossman. Crystal graph convolutional neural networks for an accurate and interpretable prediction of material properties. *Physical review letters*, 120(14):145301, 2018.

21. Kamal Choudhary and Brian DeCost. Atomistic line graph neural network for improved materials property predictions. *npj Computational Materials*, 7(1):185, 2021.

22. Yuchao Lin, Keqiang Yan, Youzhi Luo, Yi Liu, Xiaoning Qian, and Shuiwang Ji. Efficient approximations of complete interatomic potentials for crystal property prediction. In *International Conference on Machine Learning*, pages 21260–21287. PMLR, 2023.

23. Kishalay Das, Bidisha Samanta, Pawan Goyal, Seung-Cheol Lee, Satadeep Bhattacharjee, and Niloy Ganguly. Crysgnn: Distilling pre-trained knowledge to enhance property prediction for crystalline materials. In *Proceedings of the AAAI Conference on Artificial Intelligence*, volume 37, pages 7323–7331, 2023.

24. Rishikesh Magar, Yuyang Wang, and Amir Barati Farimani. Crystal twins: self-supervised learning for crystalline material property prediction. *npj Computational Materials*, 8(1):231, 2022.

25. Haomin Yu, Yanru Song, Jilin Hu, Chenjuan Guo, and Bin Yang. A crystal-specific pre-training framework for crystal material property prediction. *arXiv preprint arXiv:2306.05344*, 2023.

26. René Flukiger, Alain Paoli, and Jean Muller. Electronically 'atypical'a 15-type compounds based on chromium and molybdenum. *Solid State Communications*, 14(6):443–447, 1974.

27. Yuto Watanabe, Akira Miura, Chikako Moriyoshi, Aichi Yamashita, and Yoshikazu Mizuguchi. Observation of superconductivity and enhanced upper critical field of η-carbide-type oxide zr4pd2o. *Scientific Reports*, 13(1):22458, 2023.

28. Paolo Giannozzi, Oliviero Andreussi, Thomas Brumme, Oana Bunau, M Buongiorno Nardelli, Matteo Calandra, Roberto Car, Carlo Cavazzoni, Davide Ceresoli, Matteo Cococcioni, et al. Advanced capabilities for materials modelling with quantum espresso. *Journal of physics: Condensed matter*, 29(46):465901, 2017.

29. Claudio Zeni, Robert Pinsler, Daniel Zügner, Andrew Fowler, Matthew Horton, Xiang Fu, Sasha Shysheya, Jonathan Crabbé, Lixin Sun, Jake Smith, et al. Mattergen: a generative model for inorganic materials design. *arXiv preprint arXiv:2312.03687*, 2023.

30. Vıctor Garcia Satorras, Emiel Hoogeboom, and Max Welling. E (n) equivariant graph neural networks. In *International conference on machine learning*, pages 9323–9332. PMLR, 2021.
