# Supplementary material for: Siamese foundation models for crystal structure prediction
Source: Nat Commun. 2026 May 6;17:6103. doi: 10.1038/s41467-026-72362-3 (PMC13358074; doi:10.1038/s41467-026-72362-3)
Supplement: Supplementary file 2 — Transparent Peer Review file [file 41467_2026_72362_MOESM2_ESM.pdf]

nature portfolio

Peer Review File

Siamese Foundation Models for Crystal Structure Prediction

Corresponding Author: Professor Wenbing Huang

This file contains all reviewer reports in order by version, followed by all author rebuttals in order by version.

Version 0:

Reviewer comments:

Reviewer #1

(Remarks to the Author)
This manuscript proposes a neural network "foundation model" for crystal structure prediction. It major architecture is similar to the MatterGen work published in Zeni, C., Pinsler, R., Zügner, D. et al. A generative model for inorganic materials design. Nature 639, 624–632 (2025). https://doi.org/10.1038/s41586-025-08628-5, including the structure generator and property prediction. The only change is the backbone (changed from graph NN to graph transformer), the minor change of pretraining task (from de novo structure generation to CSP), and including unstable structures into the pretraining and relaxed structures in finetuning. The energy-guided sampling is also a plus. While these modifications are applaudable contributions, they do not represent significant intellectual contribution to the CSP field. More importantly, I feel their major performance improvements presented in Table 1 are NOT well justified and are biased (inappropriate/incorrect) evaluation procedure. Overall, due to the biased results and the lack of essential algorithmic innovation, I cannot recommend it for publication in Nature comm.

1)
Table 1 results are suspicious as the proposed models used significant more structures in their pretraining finetuning process than other baselines and the anti-data leaking approach is not enough to avoid data leaking due to the high-redundany (similarity) of structures in the OQMD dataset.

Their pretraining data are obtained from Materials Project (MP) [19] and OQMD.
In order to prevent data leakage on CSP tasks, we exclude any crystals that appear in downstream 143 benchmarks (i.e. MP-20 and MPTS-52), resulting in a deduplicated version of CrysDB that consists of 919,258 entries
For all the other baseline algorithms, they are only trained with 27136 (MP-20) and 27380 (MPTS-52) training samples.

"train/validation/test splits: 27136/160 9047/9046 for MP-20 and 27380/5000/8096 for MPTS-52"

from above information, we find that:
1) One can see that their DAO-G models are trained with almost 40 times more structures. So their improved performance is more likely due to the data increase. To allow fair comparison, they need to train the baseline algorithms also with the stable and relaxed entries from the CrysDB with 919,258 entries

2) "In addition, to prevent data leakage on CSP tasks, we exclude any crystals that appear in downstream benchmarks (i.e. MP-20 and MPTS-52), resulting in a deduplicated version of CrysDB that consists of 919,258 entries"
==
this anti-leaking strategy is not fool-proof and is actually problematic due to the highly redundant structures existent in the MP and OQMD database. This issue has lead to many over-estimated performance report in materials property prediction literature (e.g. works showing using only composition of SuperCon Db can predict the Transition temperature). This redundancy of samples can fail your leaking prevention because while you can make sure you exclude any crystals that appear in MP-20 and MPTS-52, you cannot guarantee exclude those structures that are very similar to samples in MP-20 and MPTS-52. These sister and brother structures used in the pretraining/finetuning can easily help your algorithms to outperform others.

so to ensure the performance improvement of your algorithm is not due to this data leaking, you can just pre-train the

baseline CSP algorithms first with the same CrysDB data and then finetune with the 27136 (MP-20 test) and 27380(MPTS-52 test) and then compare the result.

2)

DAO-P serves as the energy predictor, playing a dual role in relaxing the pretraining data and guiding the sampling process for DAO-G.
exponential energy loss
==need more clarification.

3)
page 7:
The most similar work to this manuscript is MatterGen.
Why you did not include Mattergen's result in Table 1 to compare with yours? this is strange and should be fixed.

4)
Can you provide the result of DAO-G only trained with the stable structures + relaxed structures? that will show why including the unstable structures for first-stage pretraining is reasonable.

page 8:
generating polymorphic structures
==
the results of polymorphic structure generation are a little bit not rigorious and has the issue of cherry-picking.
"several representative groups are selected", how many? and what is the success rate to recover all the polymorphs? how realiable is it?

Better do a rigious evaluation.

(Remarks on code availability)
The code repository is well written. with detailed instruction of how to use it.

Reviewer #2

(Remarks to the Author)
The manuscript presents a pretrain-finetune framework for crystal structure prediction (CSP), introducing a pair of Siamese foundation models—DAO-G for structure generation and DAO-P for energy prediction. The proposed models are trained on a large-scale dataset, CrysDB, and demonstrate superior performance on multiple CSP benchmarks and real-world superconductors. The work is promising for the field of materials discovery. However, the following issues should be addressed:

1.The number of entries from OQMD described in Section 2.2 appears inconsistent with the statistics shown in Figure 2a. Please verify and correct the discrepancy.

2.For the results shown in Figure 4, particularly DAO-P's performance across multiple property prediction tasks, could this superiority be due to access to a larger or more diverse training dataset? Please clarify whether the training conditions are comparable across baseline models.

3.In the second stage of DAO-G pretraining, only structures with Ehull values between 0.08 and 0.5 eV/atom were selected for relaxation. It is recommended that the authors clarify the rationale for excluding structures with Ehull > 0.5 eV/atom, and discuss whether this choice may potentially affect the model's generalization ability or structure generation performance. Additionally, it would be beneficial to include in Figure 3a a comparative experiment in which DAO-G is trained solely on the relaxed dataset from Stage II, to more intuitively demonstrate the impact of the two-stage training strategy on model performance.

4.The role of DAO-P during the generation process of DAO-G, particularly in the energy-guided sampling phase, should be explained in greater detail in the methodology section.

5.The manuscript would be significantly strengthened if the authors could demonstrate the novelty and feasibility of generated structures, for example by validating stability via DFT calculations or proposing potentially functional candidate materials generated by DAO-G.

(Remarks on code availability)
the code has been installed, but the pretrained model is not available as as it requires permission.

Reviewer #3

(Remarks to the Author)
The authors propose the pretrain-finetune framework “Diffusion-based crystal Omni” (DAO) for the task of crystal structure prediction (CSP). It comprises two models, a generative model, DAO-G, and a model for property prediction, DAO-P, which are both based on an introduced graph transformer called “Crysformer” that ensures the invariance of predictions with respect to O(3) and periodicity. To pretrain the models, the authors compile a large dataset “CrysDB” with more than 900k stable and unstable crystals that are taken from Materials Project (MP) and OQMD. The pretraining consists of multiple stages: Both models are first trained on all unstable and stable structures, then a portion of the unstable structures in the database is relaxed using the gradients of energies predicted with DAO-P, and finally DAO-G is refined using those relaxed structures and the stable structures from CrysDB to steer it towards generating low-energy crystals. Beyond relaxing a portion of the training dataset, DAO-P is further utilized to predict energies of intermediate structures during the diffusion-based generation of crystals with DAO-G, enabling energy-guided sampling.
The authors finetune their models for evaluation in multiple downstream tasks to demonstrate that they achieve state-of-the-art results in CSP. They conduct many ablation studies and present promising results on the exploration of superconductors in support of their claim that the collaboration of the two models during training and generation enhances the performance of both DAO-G and DAO-P.

Although the Figures presented in the manuscript are well crafted and the idea of utilizing a stepwise pretraining with unstable and stable crystals is generally interesting, we identified several shortcomings in the manuscript (which we provide in more detail in a list below). Most severely, the contextualization, documentation, evaluation, and discussion of the experiments often remains shallow, including contradicting information, which undermines the credibility of the results. For instance, the sources of the results for baseline methods are not specified (Table 1) or point to wrong sources (Figure 4). Thus, it is impossible to verify whether all baselines use the same setup (e.g. data splits) and whether this setup is identical to the one employed by the authors. A comparison to MatterGen[1], one of the most relevant competitors, is missing, although the code for training the MatterGen on CSP is openly available. Furthermore, it is at times hard to distinguish between novel aspects and prior work utilized in the presented framework, which heavily builds on the diffusion process proposed in DiffCSP[2]. The main differences are the elaborate pretraining procedure on CrysDB with two models and the transformer-based model architecture Crysformer (as opposed to a graph neural network in DiffCSP). The manuscript would heavily benefit from a concise description of the novel aspects and clear references to DiffCSP, whenever something is identical (e.g. the construction of edge features or the energy guidance). Similarly, the experiments and especially their discussion should be more focused on showing the benefits of these novel aspects. Instead of showing many experiments and discussing all of them rather briefly, it would be more convincing to provide a thorough analysis on the experiments that are most important to support the novel aspects and move less important findings to the Supplement, e.g. the prediction performance shown in Figure 4, which is irrelevant for CSP.
In conclusion, we recommend rejection, as the manuscript does not meet the requirements of Nature Communications. We suggest that the authors compile a majorly revised manuscript for submission to an outlet focused on materials science, such as npj Computational Materials.

You can find a list of concerns as well as questions that might help to compile an improved future version in the following:

1. The sources of the results for baseline experiments (and therefore the setup employed) are unclear.
a. The caption of Table 1 does not mention where the results for baseline methods are taken from. We checked the papers referenced for the first two methods (P-cG-SchNet[3], CDVAE[4]) and could not find the reported numbers. [3] does not deal with generation of periodic structures at all - it seems like P-cG-SchNet must be an adapted version of cG-SchNet designed in a different publication which should also be cited (where is it from, how does it work?). [4] does only report results on MP20 (but these are different from the results in Table 1) and no results on MPTS-52. There should be a clear statement for all of the baseline methods of a) where the results are exactly taken from and b) which setups were used for those experiments and whether they are the same used with DAO-G.
b. The caption of Figure 4 says that the results are taken from the original publications but there are no original publications cited inside the Figure. The text includes citations but these are not clearly assigned to the models. We checked the first citation[9] which probably belongs to SchNet but it does not contain any results on datasets containing crystals. Again, it should be clear where the numbers are taken from and how these experiments were conducted (number of train/validation/test samples, same split or not?).
c. Evaluation metrics like the match rate are only vaguely defined, not given as a formula.

2. MatterGen[1] is a strong competitor that is mentioned in the manuscript at several places, but a direct comparison to the method is missing.
a. In line 166, the authors write “While MatterGen could be modified and adapted for the CSP task here, we exclude it from our comparisons due to data leakage between its pretraining data and the test datasets”. However, MatterGen has an open source implementation and provides directions for training on custom datasets and the CSP task. Training it on CrysDB for a comparison would overcome the issue of data leakage and would be a relevant comparison.
b. It is to be determined how the separate model DAO-P for property guidance compares to the classifier free guidance approach in MatterGen.
c. In line 40 the manuscript reads “while the generative method MatterGen focuses on general-purpose crystal generation under conditions of desired chemistry, symmetry, and properties”. This is misleading, because general-purpose generation

under the condition of desired chemistry and low-energy meets the definition of CSP.

3. The method heavily relies on the methodology proposed in DiffCSP[2] but does not communicate or evaluate the similarities/differences well enough.
a. There should be a section that precisely explains the changes compared to DiffCSP. This would also give the motivation for the most important ablation studies and experiments that support how the proposed framework improves upon DiffCSP.
b. Some parts in the methods section, such as the computation of the edge features with Fourier Transformations, are only explained very vaguely. Thus, it would be hard to reproduce the method. However, many parts seem to be similar or identical to the procedures in DiffCSP. It would be better to explicitly state that the authors adhere to the methodology described in detail in DiffCSP[2] or provide more details if things differ.
c. As the authors mention in section 2.3.1 (from line 186), DAO-G without pretraining can be seen as a variant of DiffCSP. DiffCSP performs better on MP-20 and DAO-G has better results on MPTS-52. Additionally, there are experiments in the Supplements (C6 and Table S4) where the effect of pretraining DiffCSP with CrysDB is shown to only slightly improve performance, while DAO-G significantly benefits from pretraining. We think that these comparisons are very important to understand the benefits of the novel aspects in the proposed framework. Unfortunately, the analysis of these experiments are extremely shallow and important information is missing to draw meaningful conclusions. There are several interesting factors that might lead to the observations:
i. Does the graph neural network in DiffCSP have a cutoff? If yes, this might be a reason for improved performance of a transformer, since it allows interactions between all atoms. However, this would also imply a possible tradeoff in terms of scaling since a GNN with cutoff might scale sub-quadratically in the number of atoms while the transformer scales quadratically.
ii. How many parameters do the two models have? If Crysformer has many more parameters than the GNN, this would be a plausible explanation for its larger benefit from pretraining. If the number of parameters is very different, it might be better to also do experiments with similarly large models.

4. The manuscript is imprecise in addressing the data leakage that could occur when pretraining the DAO models.
a. For a fair evaluation of the CSP results in Section 2.3, it is crucial that both DAO-G and DAO-P have not been trained on any structures of MP-20 or MP-52. To address this, the authors create a deduplicated version of CrysDB. However, some passages of the manuscript are inconsistent with regard to the training of DAO-P.
i. Line 153 states that "the deduplicated version of CrysDB [is] used for pretraining DAO-G", which explicitly mentions DAO-G, but not DAO-P. On the other hand, line 170 declares, "Notably, both DAO-G and DAO-P used in this section are pretrained on the deduplicated CrysDB".
ii. In the Supplementary Information, Table S1 only contains hyperparameters for the task of training DAO-P on the whole of CrysDB, i.e. training DAO-P on CrysDB (dedup) is missing. Table S2 on the other hand also mentions a pretraining task of DAO-P on CrysDB (dedup).
iii. It remains unclear why and for which experiments DAO-P is trained on the original CrysDB instead of CrysDB (dedup)
b. In Section 2.5, the experiments on superconductors use pretrained models for finetuning on the SuperCon dataset. However, it is not clear if target structures are correctly removed from the deduplicated CrysDB database.
i. In line 288 the authors write "we finetune our pretrained models using the publicly available SuperCon dataset". This reads like the models that have been pretrained on the deduplicated CrysDB are used for the semiconductor experiments of Section 2.5. However, a structure of CsV_3Sb_5 with the same spacegroup P6/mmm as in the reference publication [5] is present in OQMD (ID 6085061, DOI: 20.500.12856/oqmd.v1-en.1514955.v1). However, because it is not included in MaterialsProject, it would not be removed through the deduplication procedure that is described in Section 2.2.
ii. On the other hand, the manuscript states in line 303: "These three superconductors are unseen during both the pretraining and finetuning processes, thereby avoiding the risk of data leakage". But the experimental setup does not mention any kind of retraining the pretrained models on CrysDB without the targeted semiconductors. For this reason it remains unclear if data leakage has been correctly avoided.

5. For pretraining of DAO-P and DAO-G the deduplicated CrysDB dataset is used to avoid data leakage. Next, unstable structures in the range of (0.08-0.5] eV/atom are relaxed based on the gradients of DAO-P energy predictions and added to the dataset for finetuning. This will likely undo parts of the datasets deduplication (i.e. add structures similar to those in the downstream datasets).
a. For a deeper understanding of the siamese approach, it would be beneficial to provide statistics on the structures that DAO-P adds to the deduplicated dataset for stage II training. I.e., how many of the structures that are removed during deduplication are added back in and what is their RMSD to the target structures? How many of the imputed structures are part of the MP-20 and MPTS-52 test sets?
b. It remains unclear if the improved performance can be addressed to the interplay between the siamese models or solely to DAO-P being able to impute structures from the test set for stage II training. How would an untrained DAO-G or other models like MatterGen (see comment 2.) perform if it was directly trained on the augmented stage II dataset?

6. Some of the design choices regarding the pretraining framework and the usage of two models are not explained, motivated, or evaluated.
a. DAO-G and DAO-P use the same architecture and have diffusion prediction heads for lattice and fractional coordinate noise, but DAO-P has an additional prediction head for energies.
i. It is unclear why the separate model DAO-G is needed if DAO-P is also trained to perform structure generation with the noise heads.
ii. If two separate models are needed, it remains unclear why DAO-P is trained to predict lattice and coordinate noise when only the energy prediction head is used.
b. In the relaxation of CrysDB structures for stage II of the DAO-G pretraining, only crystals with Ehull in the range (0.08, 0.5]

eV/Atom are considered. However, CrysDB contains molecules with Ehull up to 1.0 eV/Atom, which implies that there are still unstable structures with comparatively high Ehull (0.5, 1.0] eV/Atom in the refined dataset. Why is this the case? Can DAO-P not relax structures with Ehull > 0.5 eV/Atom? Or are the unstable structures still valuable for training? How would the performance change if these unstable structures would be removed or also relaxed?

7. The manuscript uses DFT computations with Quantum Espresso only in Section 2.5 to compare DAOs performance on finding the three superconductors to relaxation with DFT.
a. The authors do not provide any information on the Quantum Espresso settings that have been used for DFT relaxation. Figure S3 shows CsV_3Sb_5 renders of the ground truth as well as structures that are found with DAO-G and DFT. While relaxation with DFT seems to find a structure that is very close to the local minimum, the atoms still look very perturbed in comparison to the ground truth. Assuming that the ground truth structure has also been obtained through relaxation with DFT and that the authors use accurate settings for DFT, it is unclear why the Quantum Espresso relaxation is unable to further refine the structure.
b. It is unclear how unreasonable structures are defined in this experiment. Finding other (unstable) local minima is expected behaviour in structure relaxation in the physical potential energy surface, while diffusion models are trained to ignore energy barriers of local minima. However, failed DFT computations, perturbed global minimum structures and finding other local minima could also be caused by suboptimal settings of Quantum Espresso or the optimizer. It would be interesting to further analyze this.

8. Besides foundation models and generative modeling, active learning based approaches are widely used for crystal structure search [6-8]. Their independence of pre-existing databases makes them useful in settings that have not been explored before. This is not mentioned in the introduction at all.

Some minor comments:
- line 76 "of the first superconductor": Which superconductor is meant? You only explicitly mention CsV_3Sb_5 in the introduction, better explicitly state the formula, especially if it is another one.
- line 150 "across the range of 1 to 30": It should be “3 to 30”
- The binning/labeling on the x-axis in Figure 2c is a bit unintuitive, the first bin seems to capture 0.0 to 0.1 but there are 11 bins, so does the last capture 1.0 to 1.1? But the data points should all have values smaller than 1.0 according to the text.
- Is the y-axis in Figure 3c the sum of change in energy of all relaxed structures? Maybe reporting the average change in eV/atom would be more intuitive.

Literature:

[1] Zeni C, Pinsler R, Zügner D, Fowler A, Horton M, Fu X, Wang Z, Shysheya A, Crabbé J, Ueda S, Sordillo R et al. A generative model for inorganic materials design. Nature. 2025 Jan 16:1-3. https://doi.org/10.1038/s41586-025-08628-5
[2] Jiao R, Huang W, Lin P, Han J, Chen P, Lu Y, Liu Y. Crystal structure prediction by joint equivariant diffusion. Advances in Neural Information Processing Systems. 2023 Dec 15;36:17464-97.
[3] Gebauer NW, Gastegger M, Hessmann SS, Müller KR, Schütt KT. Inverse design of 3d molecular structures with conditional generative neural networks. Nature communications. 2022 Feb 21;13(1):973.
[4] Xie T, Fu X, Ganea OE, Barzilay R, Jaakkola TS. Crystal Diffusion Variational Autoencoder for Periodic Material Generation. In International Conference on Learning Representations. 2022.
[5] Ge J, Wang P, Xing Y, Yin Q, Wang A, Shen J, Lei H, Wang Z, Wang J. Charge-4 e and charge-6 e flux quantization and higher charge superconductivity in kagome superconductor ring devices. Physical Review X. 2024 Apr 1;14(2):021025.
[6] Merchant A, Batzner S, Schoenholz SS, Aykol M, Cheon G, Cubuk ED. Scaling deep learning for materials discovery. Nature. 2023 Dec 7;624(7990):80–85. https://doi.org/10.1038/s41586-023-06735-9
[7] Hessmann SSP, Schütt KT, Gebauer NWA, Gastegger M, Oguchi T, Yamashita T. Accelerating crystal structure search through active learning with neural networks for rapid relaxations. npj Computational Materials. 2025 Feb 20;11(1):44. https://doi.org/10.1038/s41524-025-01523-7
[8] Butler PWV, Hafizi R, Day GM. Machine-learned potentials by active learning from organic crystal structure prediction landscapes. J Phys Chem A. 2024 Feb 8;128(5):945–957. https://doi.org/10.1021/acs.jpca.3c07129
[9] Schütt K, Kindermans PJ, Sauceda Felix HE, Chmiela S, Tkatchenko A, Müller KR. SchNet: A continuous-filter convolutional neural network for modeling quantum interactions. Advances in neural information processing systems. 2017;30.

(Remarks on code availability)

Reviewer #4

(Remarks to the Author)
I co-reviewed this manuscript with one of the reviewers who provided the listed reports. This is part of the Nature Communications initiative to facilitate training in peer review and to provide appropriate recognition for Early Career Researchers who co-review manuscripts.

(Remarks on code availability)

Version 1:

Reviewer comments:

Reviewer #3

(Remarks to the Author)
First of all, we want to thank the authors for taking the reviewer's comments very seriously and for their detailed answers. They have addressed our concerns carefully and we think that they have drafted a significantly better manuscript following the suggestions and questions of all reviewers. Due to the newly added experiments (especially Table S1 and section 2.3.1), the causes of the improved performance of the proposed framework are now better disentangled and easier to grasp. However, as noted in our previous review, we still believe that the manuscript would be better suited for an outlet focused on materials science and we recommend transferring it, e.g. to npj Computational Materials, as the study mainly combines existing work and ideas to demonstrate slightly improved, ML-driven crystal structure prediction (CSP). As can be seen in the added experiments, the main improvement of performance stems from scaling up the model and pretraining on the large data set, which is expected and has for example previously been shown in MatterGen. The Crysformer architecture, the pretraining stage two, and the energy guidance with DAO-P further improve the performance but only slightly. So, in our view, the main contributions are:

* The authors demonstrate how a data set for pretraining can be curated using stable and unstable structures and a property predictor to obtain large models that can be fine-tuned for CSP.
* They thoroughly evaluate these models and employ them for the exploration of superconductors as an application example.

In this sense, we see the work as a well executed and interesting case study that combines and applies existing approaches rather than a significant technical advance in novel deep learning architectures or training paradigms for CSP. Thus, we believe that it does not fully meet the scope of Nature Communications, which aims to represent important advances of significance to specialists within different fields.

While we think that the manuscript now is in a good state and poses a valuable scientific contribution, we have a few open questions that should be addressed before it can be published (in the outlet deemed suitable by the editors):

1. The hyperparameters for training the baseline models in the newly added experiments are not given (the pretrained models in Table 1 and the models in Table S6).
a. The performance of SchNet and MEGNet (with and without pretraining) in Table S6 seem odd since the errors are significantly higher than the one reported in Table S5 (taken from MMPT). We understand that different implementations were used but it seems like the hyperparameter choices of the authors was far from optimal. If standard hyper-parameters of the packages were used, this should be stated and the exact package versions employed should be noted for reproducibility.
b. The same holds for the pretrained models in Table 1: It should be noted which hyperparameters were used or that standard parameters of certain code packages/bases were used and which version of these were employed for the experiments.
c. If standard hyperparameters were used for the pretrained models in Table S1, it is likely that the performance gain of the Crysformer architecture is even smaller than shown since we assume that the authors have tuned the training parameters of DAO-G.
2. We are under the impression that the data leakage with respect to the superconductor CsV_3Sb_5 is not yet properly addressed.
a. We have compared the structure from OQMD (used for pretraining) and the experimental superconductor (test target) and they are extremely similar. The main difference lies in the length of one of the axes in the lattice, i.e. the structure is slightly stretched into exactly one direction and the fractional coordinates align perfectly for all atoms except for a minor mismatch of the z-coordinates of four atoms. The lines 347-348 added by the authors ("the lattice parameters and atomic coordinates in the OQMD entry differ from those in the experimental SuperCon entry") suggest that it is a different crystal structure. However, it seems to us that both structures likely describe the same minimum but were obtained with different methods (e.g. one is more accurate than the other).
b. If both structures actually describe the same minimum, the model is only finding a version of the OQMD structure that is more accurate (assuming the experimental structures used for fine tuning have higher quality than those in OQMD used for pretraining). Then, this would not be an example of discovering a superconductor that was unknown to the model but finding more accurate versions of the pretraining data. Saying that there was no data leakage in this case would be misleading, as the structure from OQMD already is a representation of the superconductor (albeit of lower quality).
3. The gain of performance by pretraining and using the Crysformer architecture are now nicely demonstrated in section 2.3.1. However, the flow of reading is slightly disturbed, as it is sometimes unclear which version of DAO-G was used for which experiment.
a. In section 2.3, lines 192-193 it says "Besides, here we perform only first stage pretraining for our models", which sounds as if ALL experiments under section 2.3 (i.e. 2.3.1-2.3.4) use DAO-G with only stage I fine tuning. But it seems that this only holds for 2.3.1, whereas section 2.3.2 also deals with pretraining stage II.
b. It would be better if section 2.3 contained a brief outline that explains the order of the following experiments, e.g. "first, we evaluate the performance gained by pretraining our model and the baselines on the deduplicated CrysDB, then we examine the effect of using a second pretraining stage II utilizing structures relaxed with DAO-P". Similarly, it might be helpful to add a sentence at the beginning of section 2.3.2 that briefly explains that now the whole setup (stage I+II) is tested in contrast to

only stage I pretraining.
c. Table 1 says "Crysformer \+ DiffCSP (DAO-G), albeit consisting only of stage I pretraining. The rest of the paper refers to DAO-G as using both pretraining stages, so maybe you should write (DAO-G stage I) in Table 1 or remove (DAO-G) altogether.
4. We also have a few minor comments:
a. Lines 383-384 read "First, we intentionally perturbed the experimental structures before relaxation to evaluate DAO-G's robustness" but the paragraph is dealing with DFT-based structure prediction. According to the supplements, the superconductor structures obtained with DAO-G are sampled from complete noise (not from noisy test structures). So how is the noise added to structures prior to relaxation with DFT connected to the robustness of DAO-G? We do not really understand this sentence.
b. In the mathematical definitions of the evaluation metrics, using M for the total number of generated samples for both MR and RMSE is a bit confusing, since RMSE is only computed for matched structures and not over the whole set of generated samples. Furthermore, the MR formula only works for the 1-shot case, not for 20-shot generation, as the same index is used for the set of generated structures and test structures. Please make the formulas more precise.
c. We still believe that Fig 3 d should show the average change in energy instead of the sum. We don't really see a merrit in the current version of the graph since we neither know the number of samples per bin nor the average change in energy, therefore it does not tell much about the distribution of the data or the energy change. A large bar could stem from either a large number of samples or large changes in energies. If you would show the average instead, it would be clear by how much the systems have been improved on average during relaxation, depending on the initial energy.

(Remarks on code availability)

Reviewer #4

(Remarks to the Author)
I co-reviewed this manuscript with one of the reviewers who provided the listed reports. This is part of the Nature Communications initiative to facilitate training in peer review and to provide appropriate recognition for Early Career Researchers who co-review manuscripts.

(Remarks on code availability)

Reviewer #5

(Remarks to the Author)
I have been asked to review this manuscript, specifically relating to the authors responses to the previous reviewer #1, and so, comment on their responses below.

Comments on responses to reviewer 1.

1.1. I agree with the authors response, that this clarification would suggest that there is little risk of data leakage. (there will be some unavoidable, small risk of this occurring, if there are errors in the results from structure matcher).

1.2. I agree that the authors have satisfactorily responded to the reviewer's comment. While I agree with the statement that Crysformer is improving the model performance. By my understanding, this is shown by comparison of DiffCSP-large with "Our Crysformer + Diffusion) and FlowMM-large and "Our Crysformer + Flow-Matching". If this is correct, it looks like using Crysformer is adding ~ 1-3% to the performance across both of the match rates. Whereas, increasing the size of the training data does seem to have a greater impact on performance. I think that this has been reflected fairly in the revised text.

1.3. I think that this question has been answered, and the appropriate explanation added into section 4.

1.4. I am happy that this comment has been appropriately answered.

1.5. I am happy that this comment has been answered.

1.6. I thank the authors for their response to the question. Here, I find it surprising that there does not seem to be a systematic change in success rate with the number of polymorphs. Could the authors comment on this? Is it simply because of the small number of examples shown for tri and tetra morphs?

I have read through the revised manuscript and the reviewers comments & responses. In addition to those comments, I would like to on the manuscript:

In the paragraph starting at line 375, the authors refer to conventional global optimization methods and refer to their computational cost. What the authors do not discuss here is that in many conventional CSP codes, they can be used with interatomic potentials, either classical, or machine -learned. Doing this can provide speed ups of a similar order of magnitude which the authors report here. Discussion of this does need to be included in the manuscript as well. For example the CSP codes FUSE [https://pubs.rsc.org/en/content/articlelanding/2025/fd/d4fd00094c] USPEX (already referred to in the paper) and AIRSS [https://pubs.rsc.org/en/content/articlelanding/2025/fd/d4fd00134f] all can make routine use of

fast interatomic potentials, and can be used either purely with potentials, or a combination of potentials and DFT. Related to this, I wonder, could DAO-P be used as an interatomic potential with a different CSP method such as those listed above?

It would be useful for readers, if when summarizing the paper in the discussion section (line 396 onwards) to discuss the importance of the size of available training data, and it's impact in model performance. This is a significant contribution of the paper, is the assembled CrysDB database.

Given the use of a model to predict energies of crystals, it would be useful to refer to the paper by Chang Liu et al (https://www.nature.com/articles/s41524-024-01471-8), or other papers where energy is a learned property which is then used to aid in the prediction of structure.

(Remarks on code availability)

Version 2:

Reviewer comments:

Reviewer #3

(Remarks to the Author)
As in the first round of rebuttal, we want to thank the authors for taking the reviewer's comments very seriously and for their detailed answers. The authors have now clearly documented their choice of hyperparameters, ensuring the reproducibility of their work and improving the interpretability of the presented results. Furthermore, they have updated the experiments on superconductors, removing the results on CsV_3Sb_5 (which was affected by data leakage) and adding a study on Cr_6Os_2. The additional changes have further strengthened the presented results and improved the quality of the manuscript. Overall, our concerns regarding scientific best practices have been addressed and we believe that the manuscript is now suitable for publication, presenting valuable insights on AI guided materials discovery. We still believe that the technical contributions (creating and utilizing a large-scale data set for pretraining + evaluation of the method on an interesting application example) better fit in a journal that is specialized on computational materials science (e.g. npj Computational Materials). However, due to the high relevance of the application domain and the extent of the presented studies, we also consider it (borderline) in-scope for Nature Communications.

(Remarks on code availability)

Reviewer #4

(Remarks to the Author)
I co-reviewed this manuscript with one of the reviewers who provided the listed reports. This is part of the Nature Communications initiative to facilitate training in peer review and to provide appropriate recognition for Early Career Researchers who co-review manuscripts.

(Remarks on code availability)

Reviewer #5

(Remarks to the Author)
Thank you for responding to my questions. After reading your responses, I only have one outstanding point:

In the sentence starting on line 387, the authors write:

"In contrast, our DAO-G directly learns the distribution of stable crystal structures, ensuring both competitive efficiency and high accuracy."

This sentence, does not quite read correctly to me. The existing CSP methods, when using interatomic potentials can both use foundation level ML models as well as potentials trained for the specific system of interest. This means that in practice, the potentials that these codes can use are also trained on a wide range of crystal structures. e.g. When using MACE foundation potentials. And so potentially, this work is likely to have similar advantages and drawbacks as these potential models.

If left in the paper, this statement should be more in line with previous work, not in contrast. One thing that the authors can highlight differences in the training data which is available to each of their models.

(Remarks on code availability)

Open Access This Peer Review File is licensed under a Creative Commons Attribution 4.0 International License, which permits use, sharing, adaptation, distribution and reproduction in any medium or format, as long as you give appropriate credit to the original author(s) and the source, provide a link to the Creative Commons license, and indicate if changes were made.
In cases where reviewers are anonymous, credit should be given to 'Anonymous Referee' and the source.
The images or other third party material in this Peer Review File are included in the article’s Creative Commons license, unless indicated otherwise in a credit line to the material. If material is not included in the article’s Creative Commons license and your intended use is not permitted by statutory regulation or exceeds the permitted use, you will need to obtain permission directly from the copyright holder.
To view a copy of this license, visit https://creativecommons.org/licenses/by/4.0/

Responses to Reviewers' Comments for Manuscript:

Siamese Foundation Models for Crystal Structure Prediction

by
Liming Wu (Renmin University of China)
Wenbing Huang (Renmin University of China)
Rui Jiao (Tsinghua University)
Jianxing Huang (Huawei Technologies)
Liwei Liu (Huawei Technologies)
Yipeng Zhou (Huawei Technologies)
Hao Sun (Renmin University of China)
Yang Liu (Tsinghua University)
Fuchun Sun (Tsinghua University)
Yuxiang Ren (Huawei Technologies)
Ji-Rong Wen (Renmin University of China)

Note: To improve the readability of this response letter, all comments from the reviewers have been placed in clearly delineated text boxes. **In the revised manuscript, modifications are highlighted in red, while newly added content is marked in blue.**

Contents

Response to Reviewer 1 1

Response to Reviewer 2 7

Response to Reviewer 3 13

Response to Reviewer 4 30

Response to Reviewer #1

Comment 1.1

Table 1 results are suspicious as the proposed models used significant more structures in their pretraining finetuning process than other baselines and the anti-data leaking approach is not enough to avoid data leaking due to the high-redundany (similarity) of structures in the OQMD dataset.

Response 1.1:

We understand the reviewer's concern regarding potential data leakage and the structural redundancy in the OQMD dataset. We acknowledge that our explanation on the deduplication process may not have been sufficiently detailed, and we are grateful for the opportunity to clarify this.

For crystals with similar structures, two scenarios are possible: **(1) Same chemical composition.** Our data deduplication (Supplementary Note C.1) employs StructureMatcher to compute structural similarity. When a match is found between entries of the same composition, the duplicate is removed, ensuring all such similar structures are eliminated from the pretraining data. **(2) Different chemical compositions.** This scenario does not constitute data leakage. Our task is Crystal Structure Prediction (CSP), where the model learns to predict a structure from a composition. Although the pretraining dataset may contain similar structures, the model receives different compositions as input, so no information about the target structure is leaked.

In summary, our deduplication procedure effectively prevents data leakage. We hope this clarifies the rationale and robustness of our strategy. This analysis has been added to Section 2.2 (Lines 152-157) and Supplementary Note C.1 (Lines 944-952).

Comment 1.2

- One can see that their DAO-G models are trained with almost 40 times more structures. So their improved performance is more likely due to the data increase. To allow fair comparison, they need to train the baseline algorithms also with the stable and relaxed entries from the CrysDB with 919,258 entries;
- So to ensure the performance improvement of your algorithm is not due to this data leaking, you can just pretrain the baseline CSP algorithms first with the same CrysDB data and then finetune with the 27136 (MP-20 test) and 27380(MPTS-52 test) and then compare the result.

Response 1.2:

Thank you for your constructive feedback. We agree that data increase does lead to improved performance, which directly motivates our study on large-scale pretraining for CSP. Indeed, our contributions consist of two orthogonal aspects: (i) introducing large-scale pretraining for CSP and (ii) developing a stronger Transformer-based denoising model (Crysformer). To validate that our improvements are not solely attributable to increased data, we conducted additional experiments (shown in Table R1) by pretraining the baseline CSP algorithms first with the same CrysDB data following your advice.

Our implementation includes the competitively performing, open-sourced baseline models

DiffCSP [1] and FlowMM [2]. These models share a nearly identical denoising architecture, though FlowMM utilizes a more advanced generative method (flow matching vs. diffusion). Our proposed DAO method adopts diffusion which is the same generative method used by DiffCSP, but DAO incorporates a more powerful Transformer-based denoising model, *i.e.* Crysformer. To ensure a fair comparison, we scaled these baseline models to a parameter size comparable to Crysformer, resulting in the DiffCSP-large and FlowMM-large variants. Besides, here we performed only 1st-stage pretraining for our models. We have the following observations:

1. **Comparison with DiffCSP.** We observe that both pretraining and larger model size remarkably improve DiffCSP's performance, which further validates the utility of large-scale pretraining. Nonetheless, DiffCSP-large still falls short of our model (Crysformer + Diffusion) on almost all metrics, confirming the superior representational power of our proposed Transformer-based denoising model.

2. **Comparison with FlowMM.** Pretrained FlowMM demonstrates a significant performance enhancement, surpassing our original model (Crysformer + Diffusion) on the MP-20 benchmark. We attribute this to the effectiveness of its flow matching method compared to our diffusion approach. Therefore, we adopted flow matching in place of diffusion within our architecture. Interestingly, this replacement led to further gains, yielding state-of-the-art Match Rates of 71.27% on MP-20 and 35.55% on MPTS-52. While the corresponding RMSE values remain marginally behind those of FlowMM-large, we consider Match Rate to be the primary metric, as RMSE solely reflects the error of matched structures and is meaningful only when a high proportion of structures are successfully matched. The results here reaffirm that our pretraining strategy and framework are generally effective and not limited to specific cases.

Table R1: The CSP results of pretrained models.

	Model	MP-20		MPTS-52	
	Size	Match Rate (%) ↑	RMSE ↓	Match Rate (%) ↑	RMSE ↓
DiffCSP (w/o pretrain)	12.3M	51.89 ± 0.30	0.0611 ± 0.0015	12.15 ± 0.08	0.1767 ± 0.0025
FlowMM (w/o pretrain)	14.2M	61.26 ± 0.14	0.0572 ± 0.0014	16.11 ± 0.17	0.1831 ± 0.0021
DiffCSP	12.3M	50.39 ± 0.08	0.0563 ± 0.0011	18.27 ± 0.20	0.0810 ± 0.0006
DiffCSP-large	26.2M	63.93 ± 0.26	0.0425 ± 0.0011	30.77 ± 0.16	**0.0640 ± 0.0007**
FlowMM-large	25.7M	68.76 ± 0.12	**0.0386 ± 0.0011**	32.70 ± 0.65	0.0993 ± 0.0006
Our Crysformer + Diffusion	25.2M	65.60 ± 0.22	0.0411 ± 0.0005	32.52 ± 0.18	0.0731 ± 0.0008
Our Crysformer + Flow-Matching	25.2M	**71.27 ± 0.15**	0.0405 ± 0.0003	**35.55 ± 0.46**	0.1107 ± 0.0017

In summary, we conducted extensive additional pretraining experiments on CrysDB across multiple baselines, carefully matching model size and setup. These results strongly support that our performance gains are not simply due to larger training data, but rather to the effectiveness of our DAO framework and the Crysformer backbone. All updated results are included in the Section 2.3 (Lines 175-185, 195-215) and Table 1.

Comment 1.3

DAO-P serves as the energy predictor, playing a dual role in relaxing the pretraining data and guiding the sampling process for DAO-G. Exponential energy loss needs more clarification.

Response 1.3:

We thank the reviewer for pointing out this issue and agree that our previous description should be made more specific. Inspired by the energy-guided diffusion [3], our target distribution of crystal $\mathcal{M}$ is defined as $p(\mathcal{M}) \propto q(\mathcal{M})e^{-\beta\mathcal{E}(\mathcal{M})}$, where $q(\mathcal{M})$ is the data distribution and $e^{-\beta\mathcal{E}(\mathcal{M})}$ is the Boltzmann distribution with $\mathcal{E}(\mathcal{M})$ being an energy function. By adding the Boltzmann term, we encourage the generated structures to have lower energies and thus be more stable. During the forward diffusion process, we denote the *intermediate energy function* as $\mathcal{E}_t(\mathcal{M}_t)$ at each timestep t, leading to intermediate distributions $p_t(\mathcal{M}_t) \propto q_t(\mathcal{M}_t)e^{-\mathcal{E}_t(\mathcal{M}_t)}$. Therefore, in order to ensure that the reverse process produces correct samples from $p(\mathcal{M})$, it is essential to model not only the final energy $\mathcal{E}_0(\mathcal{M}_0)$ but also its intermediate counterparts $\mathcal{E}_t(\mathcal{M}_t)$. This motivates the introduction of an intermediate energy loss.

Predicting intermediate energy is *non-trivial* because $\mathcal{E}_t(\mathcal{M}_t)$ is defined as a log-expectation [3]:

$$\mathcal{E}_t(\mathcal{M}_t) = -\log \mathcal{E}_{q_0(\mathcal{M}_0|\mathcal{M}_t)}[e^{-\beta\mathcal{E}(\mathcal{M}_0)}],$$

which cannot be computed in closed form except at $t = 0$. This makes direct supervision unavailable. Moreover, naive surrogate losses (e.g., learning the energy $f_\phi(\mathcal{M}_t, t)$ by the MSE regression $\| f_\phi(\mathcal{M}_t, t) - \mathcal{E}(\mathcal{M}_0)\|_2^2$) cannot guarantee convergence to the target distribution, and often leads to degraded performance in downstream applications.

To overcome these limitations, we design our *intermediate energy loss*, which utilizes the exponential loss function with martingale policy rather than the direct MSE prediction:

$$\phi^* = \arg\min_\phi \mathbb{E}_{q_{0t}(\mathcal{M}_0,\mathcal{M}_t)}[\| e^{-f_\phi(\mathcal{M}_t,t)} - e^{-\beta\mathcal{E}_0(\mathcal{M}_0)}\|_2^2].$$

This formulation bypasses the intractable log-expectation, provably yields the exact gradient of $\mathcal{E}_t(\mathcal{M}_t)$. We presented the proof in Supplementary Notes B.3 and D.1. In this way, our design provides a principled and effective solution where existing approaches fall short.

This additional analysis has been included as Section 4.5.2 (Lines 606-629) in the revised version of the manuscript.

Comment 1.4

The most similar work to this manuscript is MatterGen. Why you did not include Mattergen's result in Table 1 to compare with yours? this is strange and should be fixed.

Response 1.4:

We are grateful to the reviewer for raising this point. The exclusion of MatterGen from Table 1 is due to its distinct objective: it is designed for de novo generation, jointly producing composition and structure, with CSP being a secondary capability. Additionally, to avoid data leakage, our methodology requires models trained on deduplicated data, whereas the released MatterGen model utilized the non-deduplicated Materials Project dataset.

Nevertheless, we acknowledge that pretraining MatterGen on our deduplicated dataset is a valuable comparison. Accordingly, we have done so using the authors' released code, scaling the model to match the size of our own. As Table R2 shows, while the retrained MatterGen performs slightly better than DAO-G on MP-20, it underperforms on the more challenging MPTS-52 dataset (e.g., MR 30.24% vs. 32.52%, RMSE 0.0741 vs. 0.0731). This indicates a limitation in scaling to larger-atom systems, which is consistent with MatterGen's original training set not including structures with more than 20 atoms.

Table R2: The CSP results of pretrained MatterGen.

	Model Size	MP-20		MPTS-52	
		Match Rate (%) ↑	RMSE ↓	Match Rate (%) ↑	RMSE ↓
MatterGen	25.3M	**66.40 ± 0.07**	**0.0392 ± 0.0004**	30.24 ± 0.11	0.0741 ± 0.0018
DAO (Stage I)	25.2M	65.60 ± 0.22	0.0411 ± 0.0005	**32.52 ± 0.18**	**0.0731 ± 0.0008**

The results are updated in the revised manuscript (Section 2.3, Lines 185-190, 215-220) and Table 1. Moreover, a comparative analysis highlighting the differences between DAO and MatterGen is provided in Supplementary Note D.2.2 (Lines 1182-1208).

Comment 1.5

Can you provide the result of DAO-G only trained with the stable structures + relaxed structures? that will show why including the unstable structures for first-stage pretraining is reasonable.

Response 1.5:

Table R3: Effect of unstable structures on model pretraining at Stage I.

	MP-20		MPTS-52	
	Match Rate (%) ↑	RMSE ↓	Match Rate (%) ↑	RMSE ↓
DAO-G (Stage I, stable)	**65.63 ± 0.07**	0.0434 ± 0.0003	31.17 ± 0.11	0.0790 ± 0.0015
DAO-G (Stage I, stable+unstable)	65.60 ± 0.22	**0.0411 ± 0.0005**	**32.52 ± 0.18**	**0.0731 ± 0.0008**

Thank you very much for this thoughtful suggestion. Introducing unstable structures in the first-stage pretraining is an important design choice that requires empirical validation. Regarding your suggestion of training with "stable + relaxed structures", we note that the relaxed structures still include some unstable entries, which makes them less suitable for isolating the precise effect of unstable data. Alternatively, we choose to compare with DAO-G trained from only stable data in the first stage. The results in Table R3 show that, except for the comparable Match Rate metric on MP-20, the full-dataset model consistently outperforms the stable-only model across other scenarios. This confirms that the incorporation of unstable structures is indeed reasonable.

Our original paper did actually included such results in Table S3, though we acknowledge that our original analysis was insufficient; we have now added a more detailed discussion in Supplementary Note C.5.1 (Lines 991-995).

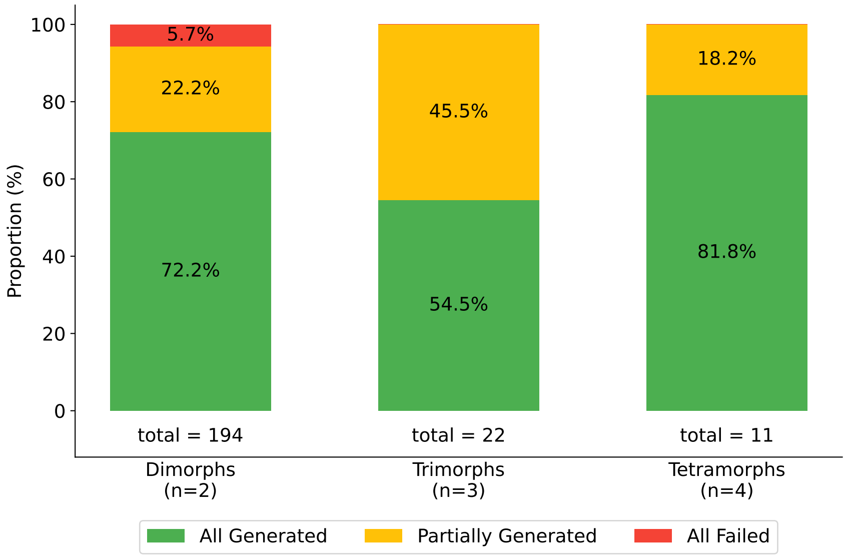

Figure R1: Generation Results Across n-Polymorphs. "All" signifies complete generation of n structures, "Partial" indicates generation of some but not all (1 to $n-1$), and "None" represents failure to generate any structure.

Comment 1.6

Generating polymorphic structures: the results of polymorphic structure generation are a little bit not rigorious and has the issue of cherry-picking. "several representative groups are selected", how many? and what is the success rate to recover all the polymorphs? how realiable is it? Better do a rigious evaluation.

Response 1.6:

We thank the reviewer for raising this important point. In the original manuscript, we only visualized several representative polymorphic structures without reporting quantitative statistics, which is insufficiently rigorous. Following your suggestion, we have conducted a more systematic evaluation.

Specifically, we considered cases with 2, 3, and 4 polymorphs. For each category, we report:

- the number of such cases in the dataset;
- the proportion of cases where all polymorphs were successfully generated;
- the proportion where part of the polymorphs (*i.e.* at least one) were recovered;
- the proportion where none of the polymorphs was recovered.

The results in Figure R1 show that: The all-success rates are 72.2%, 54.5%, and 81.8% for 2-, 3-, and 4-polymorph cases, respectively. Notably, our model successfully generates most (81.8%) of the 4-polymorph structures, demonstrating the strong capability of DAO-G in polymorphic structure generation. The occurrence of "All Failed" cases is rare, observed only at n=2 with a rate of 5.7%, and completely absent for n=3 and n=4. It further demonstrates the robustness of DAO-G.

We believe this more comprehensive evaluation addresses the concern and provides a more rigorous assessment of DAO-G in generating polymorphic structures. The added content can be found in Section 2.3.3 (Lines 252-270) and Figure 3b.

References

[1] R. Jiao, W. Huang, P. Lin, *et al.*, "Crystal structure prediction by joint equivariant diffusion," *Advances in Neural Information Processing Systems*, vol. 36, 2024.

[2] B. K. Miller, R. T. Q. Chen, A. Sriram, and B. M. Wood, "FlowMM: Generating materials with riemannian flow matching," in *Forty-first International Conference on Machine Learning*, 2024.

[3] C. Lu, H. Chen, J. Chen, H. Su, C. Li, and J. Zhu, "Contrastive energy prediction for exact energy-guided diffusion sampling in offline reinforcement learning," in *International Conference on Machine Learning*, PMLR, 2023, pp. 22 825–22 855.

Response to Reviewer #2

Comment 2.1

The number of entries from OQMD described in Section 2.2 appears inconsistent with the statistics shown in Figure 2a. Please verify and correct the discrepancy.

Response 2.1:

We thank the reviewer for catching this inconsistency. You are correct, and the number of OQMD entries in Section 2.2 should be 848,105 rather than 838,503, which corresponds to the deduplicated count. We appreciate your careful reading and correct it in the revised manuscript (Line 149).

Comment 2.2

For the results shown in Figure 4, particularly DAO-P's performance across multiple property prediction tasks, could this superiority be due to access to a larger or more diverse training dataset? Please clarify whether the training conditions are comparable across baseline models.

Response 2.2:

Thank you for your question. Figure 4 includes various baselines, both trained from scratch and pretrained on different datasets. To enable a fairer comparison as you suggested, we pretrained the SchNet [1] and MEGNet [2] baselines on our CrysDB dataset. It should be noted that the original results of these two models were reproduced from MMPT [3]. However, since MMPT is not open-sourced, we are unable to directly access its code for pretraining. As stated in the MMPT appendix, the results on the three Matbench datasets were obtained from its website, while other results were reproduced from Matformer. Although Matformer provides model parameters, it does not release the training code for these two models. As a result, different hyperparameters were used across datasets, and the lack of accessible code posed challenges for reproduction. The primary aim of this experiment is to validate the performance improvement attributable to pretraining. Therefore, it is paramount to maintain identical code implementations both before and after pretraining. For this, we used public versions from PyG[1] and DeepChem[2] to ensure differences are not due to code or configuration. We also scaled their parameters (SchNet: 25.8M, MEGNet: 25.2M) to match our DAO-P model (25.2M) closely.

[1] https://pytorchgeometric.readthedocs.io/en/latest/_modules/torch_geometric/nn/models/schnet.html#SchNet

[2] https://github.com/deepchem/deepchem/blob/master/deepchem/models/torch_models/megnet.py

Table R4: The downstream property prediction results (MAE↓) of pretrained baselines.

	MatBench			JARVIS-3D		MP		
	JDFT2D	DIELECTRIC	KVRH	Jarvis_gap	Jarvis_Ehull	Mp_Shear	Mp_Bulk	MP_gap
SchNet	67.678	0.501	0.116	0.736	0.221	0.112	0.069	0.428
SchNet (Pretrain)	66.610	0.415	0.098	0.444	0.197	0.091	0.079	0.409
MEGNet	68.251	0.387	0.081	0.446	0.159	0.109	0.073	0.377
MEGNet (Pretrain)	64.022	0.374	0.077	0.435	0.146	0.105	0.068	0.356
Our	**34.280**	**0.283**	**0.061**	**0.273**	**0.046**	**0.064**	**0.040**	**0.275**

As a result, the evaluation of these two models will differ from the original Table 4 in the original manuscript, and we make this clarification to avoid confusion. The updated results are shown in Table R4. Across eight downstream property prediction datasets, both SchNet and MEGNet generally exhibit lower MAE after pretraining (with the exception of SchNet on MP_Bulk, which shows a slight increase). However, even after pretraining on the same dataset, both models still underperform DAO-P, which highlights the strong effectiveness of our DAO-P model. We have incorporated these results into Supplementary Note C.7 (Lines 1035-1053) and Table S6.

Comment 2.3

In the second stage of DAO-G pretraining, only structures with Ehull values between 0.08 and 0.5 eV/atom were selected for relaxation. It is recommended that the authors clarify the rationale for excluding structures with Ehull > 0.5 eV/atom, and discuss whether this choice may potentially affect the model's generalization ability or structure generation performance.

Response 2.3:

We appreciate the reviewer's suggestion. We realize that our description of the relaxation process in the manuscript was insufficiently detailed, and provide a more comprehensive explanation here.

In Stage-II pretraining, we selected only structures with Ehull in the range (0.08,0.5] eV/atom for relaxation. The rationale is as follows:

- Structures with Ehull>0.5 eV/atom exhibit poor thermodynamic stability and are far from the ground-state configuration. Relaxing such structures often requires a larger optimization step size, which may adversely affect the optimization of lower-energy structures. Considering both computational efficiency and stability, we excluded structures with Ehull>0.5 eV/atom from relaxation.
- The choice of the 0.5 threshold was initially empirical, as it is nearly the midpoint of the Ehull distribution in our pretraining dataset ([0, 1.0] eV/atom).

To further validate this choice, we conducted an ablation study by varying the relaxation threshold among 0.0, 0.3, 0.5, 0.7, and 1.0—from relaxing no structure to relaxing all structures. We kept the same L-BFGS parameters in Section 4.3.2. The results (Figure R2) show that:

- Compared with no relaxation (threshold = 0.0), the threshold of 0.5 yields a higher Match Rate, suggesting that incorporating more relaxed structures in pretraining helps

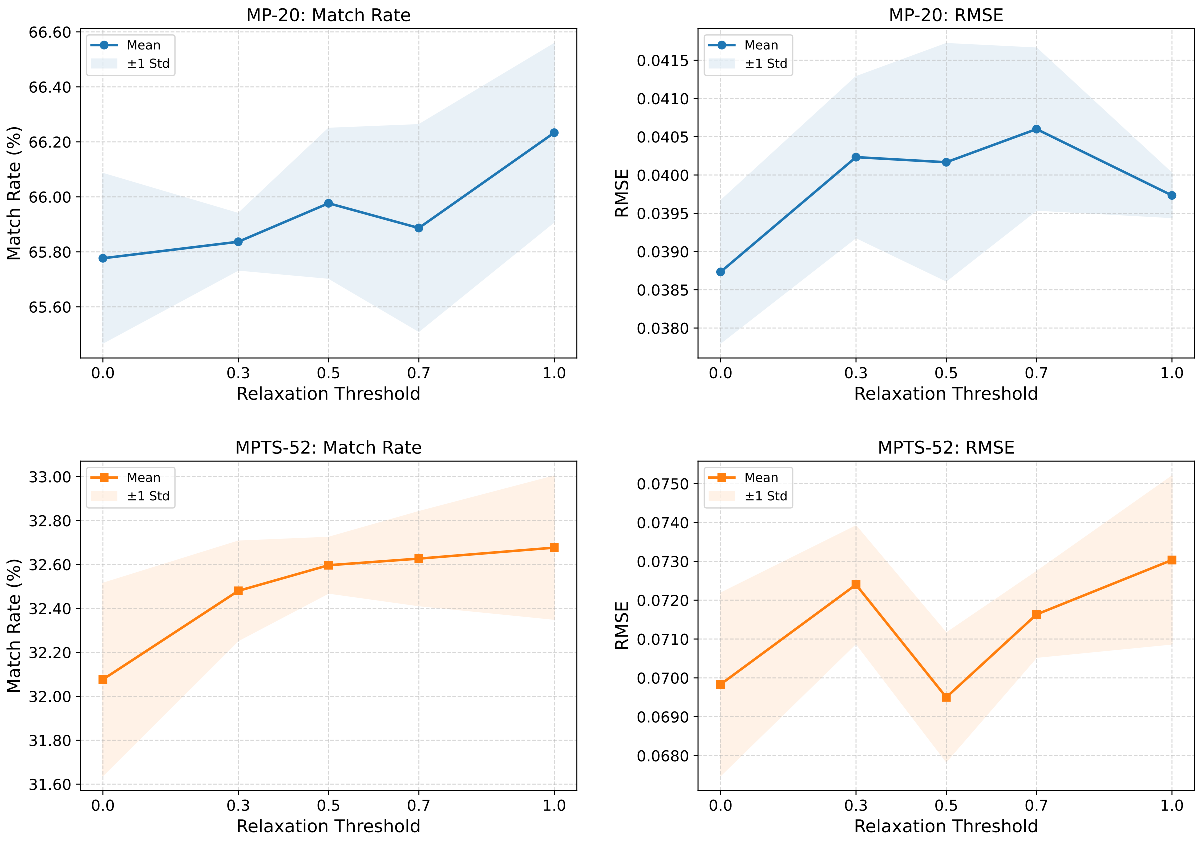

Figure R2: The influence of relaxation thresholds.

the model generate more physically plausible structures. RMSE is slightly higher, which we attribute to the model's exposure to more successfully matched structures (as Match Rate is higher), increasing the diversity of atomic coordinates after relaxation.

- For all thresholds greater than zero, the Match Rate increases on both datasets as the threshold is raised. Although a threshold of 1.0 yields the highest Match Rate, it also results in a relatively high RMSE on MPTS-52. Considering the trade-offs between all four metrics across both datasets, we found a threshold of 0.5 to offer the most balanced and reasonable performance.

Overall, a threshold of 0.5 strikes the best balance between high Match Rate, low RMSE, and reasonable relaxation cost, as it avoids excessive relaxation of very unstable structures while still improving performance. We have included a clarification in Supplementary Note C.8 (Lines 1054-1080) and Figure S2.

Comment 2.4

Additionally, it would be beneficial to include in Figure 3a a comparative experiment in which DAO-G is trained solely on the relaxed dataset from Stage II, to more intuitively demonstrate the impact of the two-stage training strategy on model performance.

Response 2.4:

As per your suggestion, we conducted an additional experiment where we pretrained DAO-G solely on the relaxed dataset from Stage II. This was done to verify whether the

improvements come mainly from two-stage pretraining. While this approach achieved reasonable results (shown in Table R5), the performance was consistently lower than that of both our Stage I–pretrained and Stage II-pretrained DAO-G. For example, compared to DAO-G (Stage I), the Match Rate dropped from 65.60% to 58.37% on MP-20, and from 32.52% to 23.78% on MPTS-52. These results indicate that our two-stage pretraining strategy, as well as retaining non-relaxed data in Stage I, is crucial for our framework's superior performance. Due to space constraints, including these results in Figure 3a would overcrowd the violin plots and obscure meaningful comparisons. Therefore, we have opted to present these findings in Supplementary Note C.5.1 (Lines 1014-1022) and Table S3.

Table R5: The performance of DAO-G pretrained with only relaxed data.

	**MP-20**		**MPTS-52**	
	**Match Rate (%) ↑**	**RMSE ↓**	**Match Rate (%) ↑**	**RMSE ↓**
DAO-G (only relaxed data)	58.37 ± 0.25	0.0605 ± 0.0019	23.78 ± 0.16	0.0964 ± 0.0004
DAO-G (Stage I)	65.60 ± 0.22	0.0411 ± 0.0005	32.52 ± 0.18	0.0731 ± 0.0008
DAO-G (Stage II)	**65.97 ± 0.22**	**0.0401 ± 0.0013**	**32.59 ± 0.10**	**0.0695 ± 0.0013**

Comment 2.5

The role of DAO-P during the generation process of DAO-G, particularly in the energy-guided sampling phase, should be explained in greater detail in the methodology section.

Response 2.5:

Nice suggestion! The role of DAO-P in the methodology section (Section 4.4) should be elaborated in greater detail. We have primarily focused on explaining the process and rationale behind energy-guided sampling. During the sampling phase of DAO-G, a pretrained DAO-P model is employed to provide energy-based guidance at each denoising step. Specifically, given the noisy intermediate structure $\mathcal{M}_t$ at timestep t , DAO-P predicts its energy $f_\phi(\mathcal{M}_t, t)$. The gradient of this energy with respect to both lattice parameters and atomic coordinates, $\nabla_{\mathcal{M}_t} f_\phi(\mathcal{M}_t, t)$, is then computed and used as a guidance term. In parallel, DAO-G predicts the noise components ϵ_L (lattice) and ϵ_F (fractional coordinates) required for reconstructing $\mathcal{M}_{t-1}$. The final update combines the predicted noise with the energy gradient, steering the structure toward lower-energy configurations. In this way, DAO-P acts as an energy oracle, ensuring that the generative trajectory of DAO-G is biased toward physically plausible, energetically favorable structures. We included these details in the revised manuscript (Section 4.4, Lines 579-588).

Comment 2.6

The manuscript would be significantly strengthened if the authors could demonstrate the novelty and feasibility of generated structures, for example by validating stability via DFT calculations or proposing potentially functional candidate materials generated by DAO-G.

Response 2.6:

Great suggestion! To further validate the stability of the structures generated by DAO-G, we performed DFT calculations to evaluate the energy above hull (Ehull) for each generated material. Since the Phase Diagram data provided by the Materials Project (MP) is based on VASP [4] with specific computational settings, we used the same tool and parameters to ensure strict consistency when calculating the total energies.

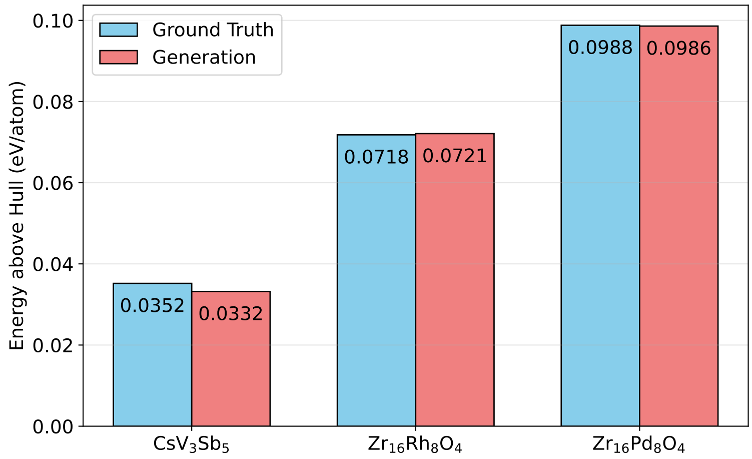

Figure R3: VASP results (Ehull) of three superconductors.

Specifically, we calculated the Ehull values for the three superconductor candidates described in Section 2.5, comparing both the experimental structures and the generated structures by DAO-G. The results (Figure R3) show that the Ehull values of the generated structures are very close to those of the experimental ones, with differences of only 0.002, 0.0003, and 0.0002 eV/atom, respectively. This demonstrates that the accuracy of DAO-G is not only reflected in the RMSE of structural reconstruction but is also robustly confirmed in terms of thermodynamic stability (Ehull). We have expanded Introduction (Lines 79-82) and Section 2.5 (Lines 364-372) to include the DFT results.

Comment 2.7

The code has been installed, but the pretrained model is not available as as it requires permission.

Response 2.7:

We apologize for the oversight. The access permissions for the pretrained model had not been properly set. This issue has now been resolved, and the pretrained model is publicly available for download via the provided Google Drive link. We appreciate your patience and thank you for bringing this to our attention.

References

[1] K. Schütt, P.-J. Kindermans, H. E. Sauceda Felix, S. Chmiela, A. Tkatchenko, and K.-R. Müller, "Schnet: A continuous-filter convolutional neural network for modeling quantum interactions," *Advances in neural information processing systems*, vol. 30, 2017.

[2] C. Chen, W. Ye, Y. Zuo, C. Zheng, and S. P. Ong, "Graph networks as a universal machine learning framework for molecules and crystals," *Chemistry of Materials*, vol. 31, no. 9, pp. 3564–3572, 2019.

[3] H. Yu, Y. Song, J. Hu, C. Guo, and B. Yang, "A crystal-specific pre-training framework for crystal material property prediction," *arXiv preprint arXiv:2306.05344*, 2023.

[4] J. Hafner, "Ab-initio simulations of materials using vasp: Density-functional theory and beyond," *Journal of computational chemistry*, vol. 29, no. 13, pp. 2044–2078, 2008.

Response to Reviewer #3

Comment 3.1

The sources of the results for baseline experiments (and therefore the setup employed) are unclear.

Response 3.1:

We sincerely appreciate the reviewer for highlighting this issue. We acknowledge that our description of the baselines lacked sufficient detail. Below, we provide a point-by-point response to address each of the questions raised, aiming to enhance clarity and understanding.

Comment 3.1-1

The caption of Table 1 does not mention where the results for baseline methods are taken from. We checked the papers referenced for the first two methods (P-cG-SchNet, CDVAE) and could not find the reported numbers. P-cG-SchNet does not deal with generation of periodic structures at all. it seems like P-cG-SchNet must be an adapted version of cG-SchNet designed in a different publication which should also be cited (where is it from, how does it work?). CDVAE does only report results on MP20 (but these are different from the results in Table 1) and no results on MPTS-52. There should be a clear statement for all of the baseline methods of a) where the results are exactly taken from and b) which setups were used for those experiments and whether they are the same used with DAO-G.

Response 3.1-1:

Thanks for bringing this to our attention. We agree that our description of the sources for the CSP baselines in Table 1 was not sufficiently clear, and we provide a more detailed clarification here. We can confirm that all baselines were evaluated under the same experimental setup as DAO-G, ensuring a fair comparison.

1. P-cG-SchNet and CDVAE: The results for these two baselines were directly taken from the DiffCSP paper [1]. You are correct that the CDVAE paper only reports results on MP-20 and does not provide results on MPTS-52. In DiffCSP, the authors extended CDVAE to MPTS-52 (Appendix B.2 in DiffCSP) and reported the corresponding results, which we adopted.

2. Remaining baselines (DiffCSP, EquiCSP, FlowMM, CrystalFlow): The results for these methods were copied directly from their respective papers. In each case, the experimental settings are consistent with those in DiffCSP.

In summary, all baseline results in Table 1 are either directly taken from the DiffCSP paper or from works that follow the same experimental protocol. This ensures that the dataset splits and other evaluation settings are identical, providing a fair comparison. We have updated the caption of Table 1.

Comment 3.1-2

The caption of Figure 4 says that the results are taken from the original publications but there are no original publications cited inside the Figure. The text includes citations but these are not clearly assigned to the models. We checked the first citation [9] which probably belongs to SchNet but it does not contain any results on datasets containing crystals. Again, it should be clear where the numbers are taken from and how these experiments were conducted (number of train/validation/test samples, same split or not?).

Response 3.1-2:

Your feedback is much appreciated. In the caption of Figure 4, we incorrectly stated "with the results directly taken from their respective papers", which may have caused confusion. We clarify the source of the results as follows:

1. Training from Scratch Models: For SchNet, CGCNN, MEGNet, ALIGNN, and Matformer, the results were directly copied from the MMPT paper [2]. The results for PotNet were taken from its original publication.

2. Pretrained Models: The results for Crystal Twins and MMPT were also taken from the MMPT paper, while the results for CrysGNN were taken from its own original publication.

Regarding the experimental settings, such as the train/validation/test split, we strictly followed the protocol in MMPT. A detailed description of the dataset splits can be found in Appendix A.1 of the MMPT paper. For PotNet and CrysGNN, we confirmed that their experimental settings were consistent with MMPT's, ensuring that including them provides a fair comparison.

Thanks again for bringing this to our attention; we've fixed the caption of Figure 4.

Comment 3.1-3

Evaluation metrics like the match rate are only vaguely defined, not given as a formula.

Response 3.1-3:

Your suggestion is very helpful for improving our paper. In the revised manuscript, we have added the explicit formulas for each evaluation metric in Section 4.6 (Lines 644-662), including Match Rate, RMSE, and MAE.

$$\text{Match\_Rate} = \frac{1}{M}\sum_{i=1}^{M} \mathbb{I}(\text{StructureMatcher}(G_i, T_i) = \text{True}), \tag{1}$$

where, M is the total number of generated samples, G_i is the i-th generated structure, T_i is the corresponding i-th ground truth structure, and $\mathbb{I}(\cdot)$ is the indicator function (1 if match succeeds, 0 otherwise). The "StructureMatcher" operates within a tolerance framework governed by three key parameters: stol (tolerance for maximum distance between atomic sites), ltol (tolerance for lattice vector lengths), and angle_tol (tolerance for lattice angles, in

degrees). It attempts to seek a one-to-one atomic correspondence that satisfies all tolerance thresholds. If such a mapping is found, it returns True; otherwise, it returns False.

$$\text{RMSE} = \frac{1}{M}\sum_{i=1}^{M}\left(\frac{1}{\sqrt[3]{V_i/N_i}}\sqrt{\sum_{j=1}^{N_i}\|\mathbf{r}_{i,j}-\mathbf{r}'_{i,j}\|^2}\right), \tag{2}$$

where, V_i is the volume of the i-th crystal, N_i is its atom number, $\mathbf{r}_{i,j}$ and $\mathbf{r}'_{i,j}$ are the Cartesian coordinates of the j-th matched atom in G_i and Structure T_i, respectively, and $\|\cdot\|$ denotes the Euclidean distance.

Note: Before computing the interatomic distances for RMSE, the structures G_i and T_i are first optimally aligned to achieve the closest structural match (automatically handled by *StructureMatcher*, requiring no manual intervention). Additionally, the RMSE is calculated only when StructureMatcher(G_i, T_i) = True; otherwise, the pair is disregarded.

$$\text{MAE} = \frac{1}{N_p}\sum_{i=1}^{N_p}|y_i - y'_i|, \tag{3}$$

where, N_p is the total number of data points, y_i is the reference value (Reverse standardization), y'_i is the predicted value (Reverse standardization), $|\cdot|$ denotes the absolute value.

Comment 3.2

MatterGen is a strong competitor that is mentioned in the manuscript at several places, but a direct comparison to the method is missing.

Response 3.2:

Thank you for your suggestion. We agree that MatterGen is an important work that merits careful discussion. To address your concerns clearly, we provide point-by-point responses and comparisons with MatterGen below.

Comment 3.2-1

In line 166, the authors write "While MatterGen could be modified and adapted for the CSP task here, we exclude it from our comparisons due to data leakage between its pretraining data and the test datasets". However, MatterGen has an open source implementation and provides directions for training on custom datasets and the CSP task. Training it on CrysDB for a comparison would overcome the issue of data leakage and would be a relevant comparison.

Response 3.2-1:

Thank you for this valuable suggestion. Following your advice, we have conducted pre-training of MatterGen on CrysDB (dedup) to avoid any data leakage issues. Particularly, we have done so using the authors' released code, scaling the model to match the size of our own. As Table R6 shows, while the retrained MatterGen performs slightly better than DAO-G

on MP-20, it underperforms on the more challenging MPTS-52 dataset (e.g., MR 30.24% vs. 32.52%, RMSE 0.0741 vs. 0.0731). This indicates a limitation in scaling to larger-atom systems, which is consistent with MatterGen's original training set not including structures with more than 20 atoms.

Table R6: The CSP results of pretrained MatterGen.

	Model Size	MP-20 Match Rate (%) ↑	MP-20 RMSE ↓	MPTS-52 Match Rate (%) ↑	MPTS-52 RMSE ↓
MatterGen	25.3M	**66.40 ± 0.07**	**0.0392 ± 0.0004**	30.24 ± 0.11	0.0741 ± 0.0018
DAO (Stage I)	25.2M	65.60 ± 0.22	0.0411 ± 0.0005	**32.52 ± 0.18**	**0.0731 ± 0.0008**

The results are updated in in the revised manuscript (Section 2.3, Lines 185-190, 215-220). Moreover, a comparative analysis highlighting the differences between DAO and MatterGen is provided in Supplementary Note D.2.2 (Lines 1182-1208).

Comment 3.2-2

It is to be determined how the separate model DAO-P for property guidance compares to the classifier free guidance approach in MatterGen.

Response 3.2-2:

This is an insightful and interesting question. While both our DAO-P model and the classifier-free guidance (CFG) approach used in MatterGen serve as mechanisms to provide guidance, they are fundamentally different in nature and purpose.

1. **Model type:** DAO-P is an explicit energy predictor, a neural network trained to estimate the energy of crystal structures (including the intermediate energies in the reverse process of diffusion). In contrast, CFG, despite its name including classifier, is not an explicit predictor and does not require a separate model to make predictions.

2. **Underlying principle:** The difference in working mechanisms is closely tied to the tasks each method addresses. Our work focuses on the Crystal Structure Prediction (CSP) task, where the goal is to generate stable crystal structures with low energy. As a result, DAO-P is designed to predict the energy and computes its gradient with respect to the structure, using this information to guide generation toward lower-energy configurations. In MatterGen, however, the goal is conditional crystal generation (e.g., conditioned on chemistry and symmetry), and CFG embeds the provided conditions into the generative model to guide the output toward satisfying those conditions.

3. **Implementation details:** DAO-P takes a crystal structure as input and outputs its predicted energy. CFG, on the other hand, encodes the condition via a lightweight adapter module, concatenates this encoding with the structure's representation, and feeds the combined input into the generative model. During training, conditions are randomly sampled with a certain probability, but there is still only one generative model plus the adapter module—no separate predictor is involved.

We have integrated the comparison into Supplementary Note D.2.2 (Lines 1182-1208).

Comment 3.2-3

In line 40 the manuscript reads "while the generative method MatterGen focuses on general-purpose crystal generation under conditions of desired chemistry, symmetry, and properties". This is misleading, because general-purpose generation under the condition of desired chemistry and low-energy meets the definition of CSP.

Response 3.2-3:

You are correct that when MatterGen is conditioned on chemistry and low-energy, it can indeed be regarded as performing the CSP task. In the manuscript, our intention was to highlight the difference in focus between DAO and existing models. DAO is designed specifically for CSP, whereas MatterGen primarily targets general-purpose crystal generation under various conditions, with CSP being only one of its possible applications.

As noted in Response 3.2-2, MatterGen incorporates different conditions into the generative process via CFG, while our approach additionally trains an energy prediction model, DAO-P, to provide guidance. DAO-P is not only capable of energy prediction but can also be finetuned for downstream property prediction tasks (as described in Section 2.4).

We have revised the corresponding description in the manuscript (Lines 41-43) to avoid any potential misunderstanding.

Comment 3.3

The method heavily relies on the methodology proposed in DiffCSP but does not communicate or evaluate the similarities/differences well enough.

Response 3.3:

You are right that our method is closely related to DiffCSP, and the differences between the two approaches need to be clarified more explicitly. To address this, we provide point-by-point responses below.

Comment 3.3-1

There should be a section that precisely explains the changes compared to DiffCSP. This would also give the motivation for the most important ablation studies and experiments that support how the proposed framework improves upon DiffCSP.

Response 3.3-1:

We fully agree with your point that adding a dedicated section to explain the differences compared to DiffCSP would benefit the paper. In the revised version, we include such a discussion in Supplementary Note D.2.1 (Lines 1160-1181), where we elaborate on both the connections and distinctions between the two approaches. The main points are as follows:

- **Relevance.** Both our DAO-G and DiffCSP target the CSP task and employ a joint diffusion process to generate both lattice and fractional coordinates.
- **Difference.** Despite these shared ideas, our framework substantially differs from DiffCSP in three aspects:

1. **Training paradigm.** DiffCSP is trained entirely from scratch, while our framework follows a pretrain–finetune paradigm. We conduct a two-stage pretraining on a large-scale crystal dataset (CrysDB, 910k structures with both structures and energies) and then finetune on the DiffCSP benchmark datasets, leading to stronger performance.
2. **Backbone architecture.** DiffCSP adopts a GNN-based backbone (similar to EGNN [3]), whereas our framework introduces a Transformer-based backbone (Crysformer), which provides stronger expressivity and generalization ability, especially under large-scale pretraining.
3. **Energy modeling.** DiffCSP briefly discusses energy optimization (Appendix G.3), where the energy at intermediate steps is supervised directly using the ground-truth energy at $t = 0$, i.e., $\mathcal{L}_{\text{DiffCSP}} = \| f_\phi(\mathcal{M}_t, t) - \mathcal{E}_0(\mathcal{M}_0)) \|_2^2$. This approach has known limitations (presented in Section 4.5.2), such as inaccurate prediction or numerical instability. In contrast, we propose a new exponential-based energy loss: $\mathcal{L}_{\text{DAO}} = \| e^{-f_\phi(\mathcal{M}_t, t)} - e^{-\mathcal{E}_0(\mathcal{M}_0)}) \|_2^2$, which we have justified theoretically as the optimal formulation (see Supplementary Note B.3).

Comment 3.3-2

Some parts in the methods section, such as the computation of the edge features with Fourier Transformations, are only explained very vaguely. Thus, it would be hard to reproduce the method. However, many parts seem to be similar or identical to the procedures in DiffCSP. It would be better to explicitly state that the authors adhere to the methodology described in detail in DiffCSP or provide more details if things differ.

Response 3.3-2:

Thank you for pointing out this shortcoming. The Fourier Transformations we used for computing the edge features indeed follow the same procedure as in DiffCSP, i.e., applying Fourier Transformations to the differences of fractional coordinates. We acknowledge that this was not clearly stated in the original manuscript, and we have now revised it accordingly (see Section 4.2, Lines 481-491).

However, the way we use edge features in our Crysformer differs from DiffCSP. While DiffCSP employs a GNN-based backbone with an EGNN-like design to ensure symmetry, we instead implement an Equivariant Graph Transformer, which provides stronger generalization and expressive power. More specifically:

1. In DiffCSP, the results of the Fourier Transformations are concatenated with the node features and then passed through the same neural network (φ_m) to compute the messages between nodes.

2. In contrast, our method applies separate neural networks to node and edge features: the networks φ_k, φ_v (introduced in Section 4.2) are used for the keys and values, while φ_e is dedicated to the edge features. The feature maps produced by φ_e are subsequently added to the outputs of φ_k and φ_v, respectively, in a corresponding element-wise fashion.

Comment 3.3-3

- Does the graph neural network in DiffCSP have a cutoff? If yes, this might be a reason for improved performance of a transformer, since it allows interactions between all atoms. However, this would also imply a possible tradeoff in terms of scaling, since a GNN with cutoff might scale sub-quadratically in the number of atoms while the transformer scales quadratically.
- How many parameters do the two models have? If Crysformer has many more parameters than the GNN, this would be a plausible explanation for its larger benefit from pretraining. If the number of parameters is very different, it might be better to also do experiments with similarly large models.

Response 3.3-3:

Thanks for your insightful questions. We would like to address both questions together.

1. Concerning the first question of whether DiffCSP applies a cutoff: DiffCSP does not use a cutoff. Its graph neural network is constructed as a fully connected graph, which also results in quadratic complexity—thus comparable to our Crysformer.

2. We sincerely appreciate your suggestion to report model sizes explicitly, as this will strengthens the completeness of our paper. In Supplementary Note C.5.2 and Table S4 of the original manuscript, we provided pretraining results for DiffCSP. The DiffCSP model has 12.3M parameters, while our DAO model has 25.6M parameters, approximately twice as many. Per your kind suggestion, to ensure a fairer comparison, we further scaled up DiffCSP (increasing the number of layers from 6 to 13), which brought its parameter count to 26.2M, slightly larger than DAO. Following the scaling, the model is pretrained on CrysDB (dedup). We observe that both pretraining and larger model size remarkably improve DiffCSP's performance, which further validates the utility of large-scale pretraining. Nonetheless, DiffCSP-large still falls short of our model (Crysformer + Diffusion) on almost all metrics, confirming the superior representational power of our proposed Transformer-based denoising model.

Table R7: The CSP results of pretrained models.

	Model Size	MP-20		MPTS-52	
		Match Rate (%) ↑	RMSE ↓	Match Rate (%) ↑	RMSE ↓
DiffCSP (w/o pretrain)	12.3M	51.89 ± 0.30	0.0611 ± 0.0015	12.15 ± 0.08	0.1767 ± 0.0025
DiffCSP	12.3M	50.39 ± 0.08	0.0563 ± 0.0011	18.27 ± 0.20	0.0810 ± 0.0006
DiffCSP-large	26.2M	63.93 ± 0.26	0.0425 ± 0.0011	30.77 ± 0.16	**0.0640 ± 0.0007**
Our Crysformer + Diffusion	25.2M	**65.60 ± 0.22**	**0.0411 ± 0.0005**	**32.52 ± 0.18**	0.0731 ± 0.0008

We thank again for these constructive comments, and these results have been added to Section 2.3 (Lines 198-200) and Table 1.

Comment 3.4

The manuscript is imprecise in addressing the data leakage that could occur when pretraining the DAO models

Response 3.4:

Thank you for raising the concern regarding potential data leakage, which is critical for ensuring the fairness and reliability of our experiments. We take this issue very seriously and will provide a point-by-point response to your questions below.

Comment 3.4-1

The manuscript is imprecise in addressing the data leakage that could occur when pretraining the DAO models. For a fair evaluation of the CSP results in Section 2.3, it is crucial that both DAO-G and DAO-P have not been trained on any structures of MP- 20 or MP-52. To address this, the authors create a deduplicated version of CrysDB. However, some passages of the manuscript are inconsistent with regard to the training of DAO-P.

1. Line 153 states that "the deduplicated version of CrysDB [is] used for pretraining DAO-G", which explicitly mentions DAO-G, but not DAO-P. On the other hand, line 170 declares, "Notably, both DAO-G and DAO-P used in this section are pretrained on the deduplicated CrysDB".

2. In the Supplementary Information, Table S1 only contains hyperparameters for the task of training DAO-P on the whole of CrysDB, i.e. training DAO-P on CrysDB (dedup) is missing. Table S2 on the other hand also mentions a pretraining task of DAO-P on CrysDB (dedup).

3. It remains unclear why and for which experiments DAO-P is trained on the original CrysDB instead of CrysDB (dedup).

Response 3.4-1:

We would like to address these three questions together, since they all pertain to the pretraining of DAO-P. We apologize for the insufficient clarity in our manuscript that has caused some confusion. Below, we provide a more detailed explanation:

In our work, DAO-P plays different roles depending on the task:

- **For the generation task (CSP in Section 2.3):** DAO-P is used to support the training of DAO-G, providing dataset relaxation and energy-guided sampling. Since the CSP task requires generating structures purely from compositions, we must ensure that neither DAO-G nor DAO-P is exposed to the crystal structures of the downstream test set in advance. Therefore, both DAO-G and DAO-P are pretrained on the deduplicated CrysDB.

- **For the property prediction tasks:** DAO-P is used as a pretrained model and is finetuned on various datasets for property prediction. In this case, the model learns structure-to-property mappings. We have carefully considered the potential risk of data leakage. Although DAO-P is pretrained on CrysDB with labels of Ehull (extracted from MP and OQMD), our downstream tasks involve a different set of labels (e.g., JARVIS_Ehull), which are not the same. Thus, pretraining DAO-P on the non-deduplicated CrysDB is acceptable, and using a larger dataset allows DAO-P to learn more robust structural representations.

With this clarification, we can now respond to your three points:

1. In line 153 (now Line 165), our intention was to emphasize that Fig. 2b–f presents statistics based on the deduplicated version of CrysDB, rather than to stress that DAO-G was specifically trained on it. To avoid confusion, we have removed the phrase "used for pretraining DAO-G." The description in line 170 (now Line 193) is accurate: in Section 2.3, both DAO-G and DAO-P are pretrained on deduplicated CrysDB.

2. We apologize for omitting the details of DAO-P on CrysDB (dedup) from Table S1, which indeed led to inconsistencies with Table S2. Apart from the dataset size, the hyperparameters are identical to those for DAO-P on CrysDB. We have now corrected Table S1 to include this information.

3. To summarize: for the CSP task, DAO-P is pretrained on deduplicated CrysDB, while for property prediction tasks, it is pretrained on the original CrysDB.

Because DAO-P serves different roles across these two categories of tasks, the pretraining datasets differ, which we acknowledge may have caused confusion in our earlier description. We will revise the manuscript to make this distinction clearer and more consistent. We are very grateful for your careful reading and constructive feedback, which helped us identify this gap in our explanation. This analysis has been incorporated to Section 4.5.1 (Lines 592-605).

Comment 3.4-2

In Section 2.5, the experiments on superconductors use pretrained models for finetuning on the SuperCon dataset. However, it is not clear if target structures are correctly removed from the deduplicated CrysDB database.

1. In line 288 the authors write "we finetune our pretrained models using the publicly available SuperCon dataset". This reads like the models that have been pretrained on the deduplicated CrysDB are used for the semiconductor experiments of Section 2.5. However, a structure of CsV3Sb5 with the same spacegroup P6/mmm as in the reference publication is present in OQMD (ID 6085061, DOI: 20.500.12856/oqmd.v1-en.1514955.v1). However, because it is not included in MaterialsProject, it would not be removed through the deduplication procedure that is described in Section

2. On the other hand, the manuscript states in line 303: "These three superconductors are unseen during both the pretraining and finetuning processes, thereby avoiding the risk of data leakage". But the experimental setup does not mention any kind of retraining the pretrained models on CrysDB without the targeted semiconductors. For this reason it remains unclear if data leakage has been correctly avoided.

Response 3.4:

Thank you very much for raising this important point regarding potential data leakage in the superconductor experiments. Since both questions relate to this concern, we address them together as follows:

1. Indeed, CsV_3Sb_5 exists in OQMD and was also present in our deduplicated pretraining dataset. However, as detailed in Supplementary C.1, our deduplication procedure relies on both composition and structural matching using StructureMatcher. The crystals are

removed only if their structures match under this procedure. In this case, although both entries share the same composition, the lattice parameters and atomic coordinates in the OQMD entry (ID 6085061) differ from those in the experimental SuperCon entry. Consequently, StructureMatcher does not classify them as equivalent, and the OQMD structure was retained. Importantly, this means that the presence of the OQMD entry does not lead to data leakage. Our double-checking confirms the validity of this result.

2. Based on the above, before conducting the superconductor experiments, we carefully verified the dataset to ensure no leakage. None of the three superconductors evaluated in Section 2.5 have matching structures in the pretraining dataset. Therefore, the DAO-G models pretrained on the deduplicated CrysDB for the CSP benchmark can be directly used for finetuning on SuperCon without retraining, while still ensuring that no data leakage occurs.

We appreciate your careful observation again. This clarity has now been added to Section 2.5 (Lines 346-349).

Comment 3.5

For pretraining of DAO-P and DAO-G the deduplicated CrysDB dataset is used to avoid data leakage. Next, unstable structures in the range of (0.08-0.5] eV/atom are relaxed based on the gradients of DAO-P energy predictions and added to the dataset for fine-tuning. This will likely undo parts of the datasets deduplication (i.e. add structures similar to those in the downstream datasets).

1. For a deeper understanding of the siamese approach, it would be beneficial to provide statistics on the structures that DAO-P adds to the deduplicated dataset for stage II training. I.e., how many of the structures that are removed during deduplication are added back in and what is their RMSD to the target structures? How many of the imputed structures are part of the MP-20 and MPTS-52 test sets?

2. It remains unclear if the improved performance can be addressed to the interplay between the siamese models or solely to DAO-P being able to impute structures from the test set for stage II training. How would an untrained DAO-G or other models like MatterGen (see comment 2.) perform if it was directly trained on the augmented stage II dataset?

Response 3.5:

Thank you for your thoughtful comments on dataset relaxation. Since both questions concern this aspect, we address them together below:

1. We acknowledge that we did not previously conduct a statistical analysis to quantify the potential overlap between the relaxed structures and the downstream test set. It is important to emphasize that even if such an overlap exists, it would not be the result of intentional data leakage. Rather, any overlap would indicate the model's robust generative capability to spontaneously produce stable structures. In addition, following your advice, we performed a detailed analysis of the structural changes during dataset relaxation. Specifically, we relaxed 429,731 structures with Ehull in the range (0.08, 0.5] eV/atom. After relaxation, we reapplied the same deduplication procedure described

in Supplementary C.1. We found that only 8 relaxed structures matched any entries in the downstream test sets (which contain 17,142 structures in total, i.e., 9,046 in MP-20 and 8,096 in MPTS-52). This proportion is extremely small, suggesting that the impact of dataset relaxation on potential data leakage is negligible. These results are added to Supplementary Note C.8 (Lines 1081-1087).

2. Regarding your second suggestion, we have conducted an additional experiment where we pretrained DAO-G solely on the relaxed dataset from Stage II. This was done to verify whether the improvements come mainly from two-stage pretraining. While this approach achieved reasonable results (shown in Table R8), the performance was consistently lower than that of both our Stage I–pretrained and Stage II-pretrained DAO-G. For example, compared to DAO-G (Stage I), the Match Rate dropped from 65.60% to 58.37% on MP-20, and from 32.52% to 23.78% on MPTS-52. These results indicate that our two-stage pretraining strategy, as well as retaining non-relaxed data in Stage I, is crucial for our framework's superior performance. Due to space constraints, including these results in Figure 3a would overcrowd the violin plots and obscure meaningful comparisons. Therefore, we have opted to present these findings in Supplementary Note C.5.1 (Lines 1014-1022) and Table S3.

Table R8: The performance of DAO-G pretrained with only relaxed data.

	MP-20		MPTS-52	
	**Match Rate (%) ↑**	**RMSE ↓**	**Match Rate (%) ↑**	**RMSE ↓**
DAO-G (only relaxed data)	58.37 ± 0.25	0.0605 ± 0.0019	23.78 ± 0.16	0.0964 ± 0.0004
DAO-G (Stage I)	65.60 ± 0.22	0.0411 ± 0.0005	32.52 ± 0.18	0.0731 ± 0.0008
DAO-G (Stage II)	**65.97 ± 0.22**	**0.0401 ± 0.0013**	**32.59 ± 0.10**	**0.0695 ± 0.0013**

Comment 3.6

Some of the design choices regarding the pretraining framework and the usage of two models are not explained, motivated, or evaluated.

Response 3.6:

Thank you for your careful reading. We agree that the motivations behind some of our design choices regarding the pretraining framework and the use of two separate models could be described more clearly. In the following responses, we address your concerns point by point.

Comment 3.6-1

DAO-G and DAO-P use the same architecture and have diffusion prediction heads for lattice and fractional coordinate noise, but DAO-P has an additional prediction head for energies.

1. It is unclear why the separate model DAO-G is needed if DAO-P is also trained to perform structure generation with the noise heads.

2. If two separate models are needed, it remains unclear why DAO-P is trained to predict lattice and coordinate noise when only the energy prediction head is used.

Response 3.6-1:

This is a very insightful question, and we agree that it is important to clarify the distinction and interplay between DAO-G and DAO-P. While they share the same backbone architecture, their training objectives and intended usages are different.

Table R9: The generative ability of DAO-G versus DAO-P on CSP tasks.

	MP-20		MPTS-52	
	**Match Rate (%) ↑**	**RMSE ↓**	**Match Rate (%) ↑**	**RMSE ↓**
DAO-P (trained on CrysDB (dedup))	63.01 ± 0.02	0.0480 ± 0.0007	29.73 ± 0.20	0.0819 ± 0.0007
DAO-G (Stage I)	**65.60 ± 0.22**	**0.0411 ± 0.0005**	**32.52 ± 0.18**	**0.0731 ± 0.0008**

1. DAO-P is indeed trained with both structure generation and energy prediction losses, so in principle it is capable of crystal structure generation. However, DAO-P is optimized under a composite objective—balancing both energy regression and denoising for structure generation—within the same model capacity as DAO-G. This trade-off inevitably weakens its ability in structure generation compared to a dedicated model. To verify this, we directly evaluated DAO-P on the CSP task. The results (Table R9) show that DAO-P underperforms DAO-G (Stage I) across all metrics. This confirms that although DAO-P can generate structures, its generative performance is compromised, which motivates training DAO-G as a separate, specialized generator. These results have now been included in the updated version of the manuscript (Table S3 and Supplementary Note C.5.1, Lines 996-1004).

2. Although only the energy prediction head of DAO-P is directly used for downstream energy prediction, the inclusion of lattice and coordinate denoising heads is still beneficial. These denoising tasks act as self-supervised signals that encourage the model to learn richer structural representations. Prior work has demonstrated the effectiveness of such diffusion-based pretraining in improving representation learning for crystal property prediction [4], and similar approaches have proven useful in other domains such as molecular property prediction [5] and semantic segmentation [6]. In our case, the incorporation of noise prediction heads enhances DAO-P's generalization capacity for energy predictions while simultaneously enabling effective finetuning for additional property prediction tasks. We have updated the analysis in our manuscript (Section 4.5.2, Lines 630-638).

Comment 3.6-2

In the relaxation of CrysDB structures for stage II of the DAO-G pretraining, only crystals with Ehull in the range (0.08, 0.5] eV/Atom are considered. However, CrysDB contains molecules with Ehull up to 1.0 eV/Atom, which implies that there are still unstable structures with comparatively high Ehull (0.5, 1.0] eV/Atom in the refined dataset. Why is this the case? Can DAO-P not relax structures with Ehull > 0.5 eV/Atom? Or are the unstable structures still valuable for training? How would the performance change if these unstable structures would be removed or also relaxed?

Response 3.6-2:

Thank you for raising this important point regarding the choice of relaxation thresholds and the role of unstable structures in Stage II pretraining. For your first two questions ("why we choose (0.08, 0.5]" and "can DAO-P not relax structures with Ehull > 0.5 eV/Atom"), we have already provided a detailed response to Reviewer #2 (Response 2.3). In summary, we found that setting the threshold at 0.5 eV/Atom achieves better balance between high match rate, low RMSE, and reasonable relaxation cost.

Table R10: Effect of unstable structures on model pretraining at Stage II.

	MP-20		MPTS-52	
	**Match Rate (%) ↑**	**RMSE ↓**	**Match Rate (%) ↑**	**RMSE ↓**
DAO-G (Stage II, stable+relax)	65.29 ± 0.14	**0.0387 ± 0.0002**	32.16 ± 0.03	**0.0685 ± 0.0011**
DAO-G (Stage II)	**65.97 ± 0.22**	0.0401 ± 0.0013	**32.59 ± 0.10**	0.0695 ± 0.0013

For your latter two questions ("are unstable structures still valuable for training?" and "how would the performance change if these unstable structures were removed or also relaxed?"), our additional experiments provide further clarification.

1. If we extend the relaxation threshold to 1.0 eV/Atom, this corresponds to the case of relaxing all unstable structures, and the results are already discussed in above response.

2. If instead we remove unstable structures entirely, i.e., only using stable + relaxed data in Stage II pretraining, the results (Table R10) indicate a trade-off between Match Rate and RMSE: performance slightly declines in terms of Match Rate while improving in terms of RMSE. For instance, compared to using the full dataset, the Match Rate decreases from 65.97% to 65.29% on MP-20 and from 32.59% to 32.16% on MPTS-52, while the RMSE shows a corresponding improvement. This suggests that incorporating unstable (unrelaxed) structures contributes to the generation of more reasonable structures (as reflected in the higher Match Rate), even though it leads to a marginal degradation in RMSE.

Therefore, retaining unstable data in Stage II proves beneficial. We hypothesize that this effect arises because including unstable structures in Stage I has already been shown to improve generalization (see Response 2.4). Retaining them in Stage II helps mitigate catastrophic forgetting and further stabilizes the learned representations. We have updated these discussions in the revised manuscript (Supplementary Note C.5.1, Lines 1005-1013).

Comment 3.7

The manuscript uses DFT computations with Quantum Espresso only in Section 2.5 to compare DAOs performance on finding the three superconductors to relaxation with DFT.

Response 3.7:

Below, we provide point-by-point responses to your questions regarding the DFT computations and the superconductor experiments.

Comment 3.7-1

The authors do not provide any information on the Quantum Espresso settings that have been used for DFT relaxation. Figure S3 shows CsV_3Sb_5 renders of the ground truth as well as structures that are found with DAO-G and DFT. While relaxation with DFT seems to find a structure that is very close to the local minimum, the atoms still look very perturbed in comparison to the ground truth. Assuming that the ground truth structure has also been obtained through relaxation with DFT and that the authors use accurate settings for DFT, it is unclear why the Quantum Espresso relaxation is unable to further refine the structure.

Response 3.7-1:

Thank you for pointing this out. The Quantum Espresso (QE) settings used for DFT relaxation were indeed provided in the Supplementary Materials (Table S8), including parameters such as *ecutwfc*, *conv_thr*, and *cell_dynamics*. We realize that our description in the main text may have been too brief, which could have made this information difficult to locate at first.

The CsV_3Sb_5 structure in Figure S3 corresponds to experimental data. The mismatch you observe between the DFT-relaxed structure and the ground truth, despite a relatively small RMSE of 0.0516, can be explained by two factors: (1) To test the robustness of DAO-G, we deliberately perturbed the experimental structure before relaxation (10% noise for abc, 10° noise for $\alpha\beta\gamma$, 0.1 noise for $\boldsymbol{F}$, introduced in Supplementary Note C.10.2). Such perturbations can lead the DFT relaxation to converge to a nearby local minimum rather than exactly recovering the experimental structure, causing a visible discrepancy even though the numerical error is small. (2) DFT relaxation intrinsically corresponds to structures at 0 K, while the experimental ground-truth structures are measured at finite temperatures. For example, the superconductor CsV_3Sb_5 is measured at 2.3 K. This temperature discrepancy can introduce inevitable deviations, even if the DFT calculations themselves are highly accurate.

We appreciate your comment, as it highlights an important detail. In the revised version (Section 2.5, Lines 380-390), we expanded our explanation to clarify these points more thoroughly.

Comment 3.7-2

It is unclear how unreasonable structures are defined in this experiment. Finding other (unstable) local minima is expected behaviour in structure relaxation in the physical potential energy surface, while diffusion models are trained to ignore energy barriers of local minima. However, failed DFT computations, perturbed global minimum structures and finding other local minima could also be caused by suboptimal settings of Quantum Espresso or the optimizer. It would be interesting to further analyze this.

Response 3.7-2:

Thank you very much for this suggestion. We realize that we did not clearly define what we mean by unreasonable structures, and we appreciate the opportunity to clarify. Importantly, in our setting, *unreasonable structures* are defined as those that fail to match ground-truth crystals according to StructureMatcher (noted in Section 2.5, Lines 380-381).

As you correctly noted, DFT relaxations may converge to alternative local minima on the potential energy surface, which is physically expected. Interestingly, this highlights one of the strengths of our diffusion-based generative model: it is capable of proposing candidate structures that are closer to the global minimum, whereas DFT relaxation alone may get trapped in a nearby local basin, depending on the quality of the initial input structure.

We fully agree that more advanced global optimization approaches (e.g., USPEX [7], CALYPSO [8]), combining DFT with genetic algorithms or evolutionary search, could potentially further reduce the probability of being trapped in local minima. However, these methods come with significantly higher computational costs, whereas our approach provides an efficient, end-to-end way of generating high-quality candidate structures.

In Section 2.5 (Lines 381-390), we have expanded the discussion to make these points more explicit.

Comment 3.8

Besides foundation models and generative modeling, active learning based approaches are widely used for crystal structure search. Their independence of pre-existing databases makes them useful in settings that have not been explored before. This is not mentioned in the introduction at all.

Response 3.8:

Thank you for highlighting this important point and for pointing us to the relevant references. We agree that active learning-based approaches constitute an important line of work in crystal structure search, especially due to their independence from pre-existing databases, which makes them particularly suitable for unexplored chemical and structural spaces. We acknowledge that this aspect was overlooked in our introduction. Following your suggestion, we have revised the introduction (Lines 44-46) to include a discussion of active learning-based methods alongside foundation models and generative approaches, so as to provide a more complete overview of the field.

Comment 3.9

Some minor comments.

Response 3.9:

Thank you very much for raising these valuable points. We have carefully considered each suggestion and have revised the paper accordingly. Below, we provide a detailed, point-by-point response to address all the issues you raised.

Comment 3.9-1

Line 76 "of the first superconductor": Which superconductor is meant? You only explicitly mention CsV_3Sb_5 in the introduction, better explicitly state the formula, especially if it is another one.

Response 3.9-1:

Thank you for pointing this out. The “first superconductor” mentioned refers to CsV_3Sb_5. We agree that explicitly stating the chemical formula makes the text clearer and avoids ambiguity. Following your suggestion, we have revised the manuscript accordingly (Line 84).

Comment 3.9-2

Line 150 "across the range of 1 to 30": It should be "3 to 30".

Response 3.9-2:

Thank you for catching this typo. You are correct that the atom number range should be from 3 to 30. We have corrected this in the revised manuscript (Lines 147-148).

Comment 3.9-3

The binning/labeling on the x-axis in Figure 2c is a bit unintuitive, the first bin seems to capture 0.0 to 0.1 but there are 11 bins, so does the last capture 1.0 to 1.1? But the data points should all have values smaller than 1.0 according to the text.

Response 3.9-3:

Thank you for pointing out the issue with Figure 2c. You are correct that the Ehull values lie within the range [0, 1]. The x-axis should consist of 10 bins, and the labeling error was caused by a plotting parameter setting. We have corrected this in the revised version (Figure 2c), where each x-axis tick now denotes the bin center.

Comment 3.9-4

Is the y-axis in Figure 3c the sum of change in energy of all relaxed structures? Maybe reporting the average change in eV/atom would be more intuitive.

Response 3.9-4:

You are correct that the y-axis in Figure 3c represents the sum of the Ehull changes for all relaxed structures within each bin. We also considered reporting the average change in eV/atom. However, since the number of data points varies significantly across bins, using the average would obscure this difference. By reporting the sum, we believe the plot conveys richer

information about both the magnitude of change and the data distribution. Nevertheless, we appreciate your suggestion and will clarify this point in the revised version.

References

[1] R. Jiao, W. Huang, P. Lin, *et al.*, "Crystal structure prediction by joint equivariant diffusion," *Advances in Neural Information Processing Systems*, vol. 36, 2024.

[2] H. Yu, Y. Song, J. Hu, C. Guo, and B. Yang, "A crystal-specific pre-training framework for crystal material property prediction," *arXiv preprint arXiv:2306.05344*, 2023.

[3] V. G. Satorras, E. Hoogeboom, and M. Welling, "E (n) equivariant graph neural networks," in *International conference on machine learning*, PMLR, 2021, pp. 9323–9332.

[4] Z. Song, Z. Meng, and I. King, "A diffusion-based pre-training framework for crystal property prediction," in *Proceedings of the AAAI Conference on Artificial Intelligence*, vol. 38, 2024, pp. 8993–9001.

[5] S. Zaidi, M. Schaarschmidt, J. Martens, *et al.*, "Pre-training via denoising for molecular property prediction," *arXiv preprint arXiv:2206.00133*, 2022.

[6] E. A. Brempong, S. Kornblith, T. Chen, N. Parmar, M. Minderer, and M. Norouzi, "Denoising pretraining for semantic segmentation," in *Proceedings of the IEEE/CVF conference on computer vision and pattern recognition*, 2022, pp. 4175–4186.

[7] C. W. Glass, A. R. Oganov, and N. Hansen, "Uspex—evolutionary crystal structure prediction," *Computer physics communications*, vol. 175, no. 11-12, pp. 713–720, 2006.

[8] Y. Wang, J. Lv, L. Zhu, and Y. Ma, "Calypso: A method for crystal structure prediction," *Computer Physics Communications*, vol. 183, no. 10, pp. 2063–2070, 2012.

Response to Reviewer #4

Comment 4.1

I co-reviewed this manuscript with one of the reviewers who provided the listed reports. This is part of the Nature Communications initiative to facilitate training in peer review and to provide appropriate recognition for Early Career Researchers who co-review manuscripts.

Response 4.1:

Thank you for your time and effort in reviewing our manuscript. We sincerely appreciate your participation in the peer-review process as part of the Nature Communications initiative to support early-career researchers. Your feedback, along with the comments from the other reviewers, has been invaluable in improving our work. We have carefully addressed all the points raised in the previous reports and hope that our revisions meet your expectations. Once again, we are grateful for your constructive role in this process.

Responses to Reviewers' Comments for Manuscript:

Siamese Foundation Models for Crystal Structure Prediction

by
Liming Wu (Renmin University of China)
Wenbing Huang (Renmin University of China)
Rui Jiao (Tsinghua University)
Jianxing Huang (Huawei Technologies)
Liwei Liu (Huawei Technologies)
Yipeng Zhou (Huawei Technologies)
Hao Sun (Renmin University of China)
Yang Liu (Tsinghua University)
Fuchun Sun (Tsinghua University)
Yuxiang Ren (Huawei Technologies)
Ji-Rong Wen (Renmin University of China)

Note: To improve the readability of this response letter, all comments from the reviewers have been placed in clearly delineated text boxes. **In the revised manuscript, modifications are highlighted in red, while newly added content is marked in blue.**

Contents

Response to Reviewer 3 1

Response to Reviewer 4 11

Response to Reviewer 5 12

Response to Reviewer #3

Comment 3.1

The hyperparameters for training the baseline models in the newly added experiments are not given (the pretrained models in Table 1 and the models in Table S6).

Response 3.1:

Thank you for highlighting this important point. We fully agree that reporting training hyperparameters is crucial for reproducibility. Below, we provide a point-by-point response to address this issue.

Comment 3.1-1

The performance of SchNet and MEGNet (with and without pretraining) in Table S6 seem odd since the errors are significantly higher than the one reported in Table S5 (taken from MMPT). We understand that different implementations were used but it seems like the hyperparameter choices of the authors was far from optimal. If standard hyper-parameters of the packages were used, this should be stated and the exact package versions employed should be noted for reproducibility.

Response 3.1-1:

Thank you for this very helpful comment. We agree that the initially reported results for SchNet and MEGNet in Table S6 could raise concerns regarding suboptimal hyperparameter choices and reproducibility.

In the previous revision, the results in Table S6 were obtained by training SchNet and MEGNet using the same hyperparameter settings as DAO-P, which may not be necessarily optimal for these architectures. Following your suggestion, we have now performed a dedicated hyperparameter grid search for both SchNet and MEGNet. Specifically, for the MP_gap dataset (which is relatively large), we search over learning rates $\{0.5, 1\} \times 10^{-3}$ and weight decay values $\{0.5, 1\} \times 10^{-4}$. For the other datasets, we use a broader search space with learning rates $\{0.1, 0.5, 1\} \times 10^{-3}$ and weight decay values $\{0.05, 0.1, 0.5, 1\} \times 10^{-3}$.

For comparison, we present the original and hyperparameter-tuned results in Table R1 and Table R2, respectively. As anticipated, most results improve after proper hyperparameter optimization. Importantly, these updated results do not change our main conclusions: pre-training consistently outperforms training from scratch for both SchNet and MEGNet, while DAO-P still achieves the best overall performance.

We appreciate this suggestion, as it significantly improves the rigor, fairness, and reproducibility of our baseline comparisons. The best-performing results after this hyperparameter tuning have now been updated in Table S6, and the corresponding optimal hyperparameter settings are explicitly reported in Table S7. Moreover, as our primary focus is on CSP, we have streamlined the main text and moved the original Section 2.4 (Accurate Crystal Property Prediction via the Finetuned DAO-P) to the Supplementary Information, where it has been merged with Supplementary Note C.7 to provide a dedicated and cohesive discussion of DAO-P's performance in crystal property prediction.

Table R1: The downstream property prediction results (MAE↓) of the pretrained baselines, **before hyperparameters tuning.**

	MatBench			JARVIS-3D		MP		
	**JDFT2D**	**DIELECTRIC**	**KVRH**	**Jarvis_gap**	**Jarvis_Ehull**	**Mp_Shear**	**Mp_Bulk**	**MP_gap**
SchNet	67.678	0.501	0.116	0.736	0.221	0.112	0.069	0.428
SchNet (Pretrain)	66.610	0.415	0.098	0.444	0.197	0.091	0.079	0.409
MEGNet	68.251	0.387	0.081	0.446	0.159	0.109	0.073	0.377
MEGNet (Pretrain)	64.022	0.374	0.077	0.435	0.146	0.105	0.068	0.356
Our	**34.280**	**0.283**	**0.061**	**0.273**	**0.046**	**0.064**	**0.040**	**0.275**

Table R2: The downstream property prediction results (MAE↓) of the pretrained baselines, **after hyperparameters tuning.**

	MatBench			JARVIS-3D		MP		
	**JDFT2D**	**DIELECTRIC**	**KVRH**	**Jarvis_gap**	**Jarvis_Ehull**	**Mp_Shear**	**Mp_Bulk**	**MP_gap**
SchNet	65.912	0.417	0.104	0.635	0.205	0.106	0.069	0.412
SchNet (Pretrain)	63.503	0.402	0.095	0.444	0.177	0.091	0.072	0.379
MEGNet	67.143	0.360	0.081	0.429	0.159	0.105	0.073	0.351
MEGNet (Pretrain)	54.713	0.349	0.073	0.403	0.144	0.101	0.068	0.338
Our	**34.280**	**0.283**	**0.061**	**0.273**	**0.046**	**0.064**	**0.040**	**0.275**

Comment 3.1-2

The same holds for the pretrained models in Table 1: It should be noted which hyperparameters were used or that standard parameters of certain code packages/bases were used and which version of these were employed for the experiments.

Response 3.1-2:

Thank you for this important suggestion, and we apologize for the earlier lack of clarity regarding the hyperparameter settings of the baseline models in Table 1.

For the pretrained baselines, we followed the training protocols and hyperparameter settings described in the original papers whenever applicable. To further ensure robustness and fairness, and in response to the reviewer's concerns, we additionally conduct a learning-rate sensitivity analysis around the default values reported in the original implementations. Concretely, we perform learning-rate searches in the following ranges:

- DiffCSP and DiffCSP-Large: $lr \in \{0.2, 0.3, 0.5, 1\} \times 10^{-4}$;
- MatterGen: $lr \in \{0.1, 0.5, 1, 5\} \times 10^{-4}$;
- FlowMM-Large and FlowMM-Crysformer: $lr \in \{1, 2, 3, 4\} \times 10^{-4}$;

The original and hyperparameter-tuned results are presented in Table R3 and Table R4, respectively. Our Crysformer + Flow-Matching model shows a notable improvement (with MR increasing from 71.27% to 74.17% on MP-20 and from 35.55% to 42.01% on MPTS-52), while other baselines exhibit less significant changes. This further underscores the effectiveness of the Crysformer architecture.

We thank the reviewer for this suggestion again, which help strengthen the rigor of our experimental evaluation. The best-performing configurations identified through these hyperparameter searches are now reported in the updated Table 1 and the corresponding hyperparameters are presented in Supplementary Note C3.2 (Lines 977-987).

Table R3: The CSP results of pretrained models, **before hyperparameter tuning.**

	Model Size	MP-20 Match Rate (%) ↑	MP-20 RMSE ↓	MPTS-52 Match Rate (%) ↑	MPTS-52 RMSE ↓
DiffCSP (w/o pretrain)	12.3M	51.89 ± 0.30	0.0611 ± 0.0015	12.15 ± 0.08	0.1767 ± 0.0025
FlowMM (w/o pretrain)	14.2M	61.26 ± 0.14	0.0572 ± 0.0014	16.11 ± 0.17	0.1831 ± 0.0021
DiffCSP	12.3M	50.39 ± 0.08	0.0563 ± 0.0011	18.27 ± 0.20	0.0810 ± 0.0006
DiffCSP-large	26.2M	63.93 ± 0.26	0.0425 ± 0.0011	30.77 ± 0.16	**0.0640 ± 0.0007**
FlowMM-large	25.7M	68.76 ± 0.12	**0.0386 ± 0.0011**	32.70 ± 0.65	0.0993 ± 0.0006
MatterGen	25.3M	66.40 ± 0.07	0.0392 ± 0.0004	30.24 ± 0.11	0.0741 ± 0.0018
Our Crysformer + Diffusion	25.2M	65.60 ± 0.22	0.0411 ± 0.0005	32.52 ± 0.18	0.0731 ± 0.0008
Our Crysformer + Flow-Matching	25.2M	**71.27 ± 0.15**	0.0405 ± 0.0003	**35.55 ± 0.46**	0.1107 ± 0.0017

Table R4: The CSP results of pretrained models, **after hyperparameter tuning.**

	Model Size	MP-20 Match Rate (%) ↑	MP-20 RMSE ↓	MPTS-52 Match Rate (%) ↑	MPTS-52 RMSE ↓
DiffCSP (w/o pretrain)	12.3M	51.89 ± 0.30	0.0611 ± 0.0015	12.15 ± 0.08	0.1767 ± 0.0025
FlowMM (w/o pretrain)	14.2M	61.26 ± 0.14	0.0572 ± 0.0014	16.11 ± 0.17	0.1831 ± 0.0021
DiffCSP	12.3M	51.23 ± 0.02	0.0552 ± 0.0010	18.50 ± 0.29	0.0825 ± 0.0007
DiffCSP-large	26.2M	64.04 ± 0.02	0.0433 ± 0.0009	30.77 ± 0.16	**0.0640 ± 0.0007**
FlowMM-large	25.7M	69.95 ± 0.14	0.0378 ± 0.0005	33.78 ± 0.43	0.0951 ± 0.0024
MatterGen	25.3M	67.40 ± 0.12	**0.0332 ± 0.0014**	30.28 ± 0.19	0.0703 ± 0.001
Our Crysformer + Diffusion	25.2M	65.60 ± 0.22	0.0411 ± 0.0005	32.52 ± 0.18	0.0731 ± 0.0008
Our Crysformer + Flow-Matching	25.2M	**74.17 ± 0.21**	0.0400 ± 0.0007	**42.01 ± 0.20**	0.1083 ± 0.0005

Comment 3.1-3

If standard hyperparameters were used for the pretrained models in Table S1, it is likely that the performance gain of the Crysformer architecture is even smaller than shown since we assume that the authors have tuned the training parameters of DAO-G.

Response 3.1-3:

We thank the reviewer for raising this concern, which allows us to further clarify the role of hyperparameter tuning and the contribution of the Crysformer architecture.

As discussed in Response 3.1-2, we have performed explicit hyperparameter tuning for the pretrained baseline models, rather than relying solely on their default settings. Notably, the effectiveness of Crysformer is consistently maintained. In particular, we highlight three pairs of comparisons:

1. DiffCSP-Large vs. Our Crysformer + Diffusion, where both models are diffusion-based and trained on the same large-scale CrysDB data. Replacing the GNN-style backbone

with Crysformer leads to consistent improvements across metrics: MR increases from 64.04% to 65.60% on MP-20, and from 30.99% to 32.52% on MPTS-52.

2. FlowMM-Large vs. Our Crysformer + Flow-Matching, which provides an even stronger and cleaner comparison. Under the same flow-matching framework, introducing Crysformer yields substantial gains: MR improves from 69.95% to 74.17% on MP-20, and from 33.78% to 42.01% on MPTS-52.

3. MatterGen vs. Our methods, which aligns with the previous finding. Although the retrained MatterGen performs better than our Crysformer + Diffusion on MP-20, it underperforms on the more challenging MPTS-52 dataset (MR: 30.28% vs. 32.52%). Notably, our Crysformer + Flow-Matching surpasses MatterGen on both datasets in terms of MR.

These improvements are observed across both diffusion-based and flow-based generative paradigms, demonstrating that the performance gains are attributable to the Crysformer architecture itself rather than to hyperparameter tuning or training protocol differences. This consistent trend strongly supports the conclusion that Crysformer provides a more expressive and scalable backbone for crystal structure generation.

Comment 3.2

We are under the impression that the data leakage with respect to the superconductor CsV3Sb5 is not yet properly addressed.

Response 3.2:

Thank you for your comment. We acknowledge that our previous description of CsV_3Sb_5 was inaccurate. We have clarified this issue and, to avoid any potential confusion, replaced it with a new superconductor Cr_6Os_2 in the experiments. Detailed point-by-point responses are provided as follows.

Comment 3.2-1

We have compared the structure from OQMD (used for pretraining) and the experimental superconductor (test target) and they are extremely similar. The main difference lies in the length of one of the axes in the lattice, i.e. the structure is slightly stretched into exactly one direction and the fractional coordinates align perfectly for all atoms except for a minor mismatch of the z-coordinates of four atoms. The lines 347-348 added by the authors ("the lattice parameters and atomic coordinates in the OQMD entry differ from those in the experimental SuperCon entry") suggest that it is a different crystal structure. However, it seems to us that both structures likely describe the same minimum but were obtained with different methods (e.g. one is more accurate than the other).

Response 3.2-1:

Thank you very much for your careful and insightful analysis. We agree with your observation that the OQMD structure and the experimental CsV_3Sb_5 structure are indeed highly similar and likely correspond to the same underlying minimum, obtained using different methodologies.

In our previous response, we erroneously concluded the two structures were distinct. This mistake stemmed from a difference in data format: the experimental CIF file for the superconductor contained oxidation state annotations, while the OQMD structure used for pretraining did not. When using "StructureMatcher", these annotations were treated as distinct species, preventing a correct match. We thank you for identifying this issue. After correcting it, we have re-examined the other two superconductors in Section 2.4 and confirmed they are not affected.

To eliminate any residual ambiguity, we have substituted CsV_3Sb_5 with a different superconductor, Cr_6Os_2 [1], in our experiments. This structure, sourced from the MPDS database (`https://mpds.io/`) (ID: S548506), is provided as a CIF file in the Supplementary Information. As demonstrated in the revised Figure 4, although the Tc prediction of Cr_6Os_2 is not as accurate as the remaining two superconductors, the structure generated by DAO-G is much closer to the experimental ground truth (RMSE = 0.0012), achieves a 100% Match Rate under 20-shot generation, and can be generated more efficiently ($\approx$ 1.5 minutes, compared to $\approx$ 5 minutes for CsV_3Sb_5).

It is also important to clarify the data leakage check for Cr_6Os_2. Both the Materials Project and OQMD contain multiple entries with this chemical formula but distinct crystal structures. Among these, structures like mp-416 and oqmd-29569 are very similar to the experimental Cr_6Os_2 and are thermodynamically stable (Ehull $\approx$ 0.026). Crucially, these specific entries were explicitly removed from our pretraining data during deduplication against the MP-20 and MPTS-52 benchmarks. The remaining Cr_6Os_2 entries in MP and OQMD are structurally dissimilar and unstable (Ehull $>$ 0.08), as summarized in Table R5; including these in pretraining does not constitute data leakage under our crystal structure prediction setting.

Table R5: The data-id and corresponding Ehull of Cr_6Os_2 in our pretraining dataset.

ID	oqmd-349259	oqmd-324773	oqmd-312965	oqmd-302423	mp-1226294
Ehull (eV/atom)	0.223	0.255	0.106	0.226	0.383

Interestingly, although the model encountered unstable Cr_6Os_2 structures during pretraining, it does not show a bias toward reproducing them. Instead, DAO-G generates the stable superconducting structure, with a DFT-calculated Ehull of 0.02918, closely matching the experimental value (0.02916). This indicates that DAO-G is not merely memorizing training examples but has learned a meaningful distribution over stable crystal structures conditioned on composition, even when unstable polymorphs exist in the training data.

We have updated the manuscript accordingly to reflect these corrections and clarifications throughout our paper (*e.g.*, Abstract, Introduction, Section 2.4, Figure 4). We sincerely thank you again for this very helpful comment, which significantly improved both the correctness and clarity of our work.

Comment 3.2-2

If both structures actually describe the same minimum, the model is only finding a version of the OQMD structure that is more accurate (assuming the experimental structures used for fine tuning have higher quality than those in OQMD used for pretraining). Then, this would not be an example of discovering a superconductor that was unknown to the model but finding more accurate versions of the pretraining data. Saying that there was no data leakage in this case would be misleading, as the structure from OQMD already is a representation of the superconductor (albeit of lower quality).

Response 3.2-2:

In Response 3.2-1, we acknowledged this issue. Accordingly, we have added a new structure, Cr_6Os_2, which further demonstrates the capability of our DAO-G model in predicting real-world superconducting structures. Thank you for this comment.

Comment 3.3

The gain of performance by pretraining and using the Crysformer architecture are now nicely demonstrated in section 2.3.1. However, the flow of reading is slightly disturbed, as it is sometimes unclear which version of DAO-G was used for which experiment.

Response 3.3:

Thank you for your positive feedback on Section 2.3.1 and for pointing out this clarity issue. We agree that the readability can be affected if it is not clear which version of DAO-G is used in each experiment. Below, we have revised the manuscript in a point-by-point manner to explicitly specify the exact DAO-G variant. We believe these changes significantly improve the clarity and coherence of the presentation.

Comment 3.3-1

In section 2.3, lines 192-193 it says "Besides, here we perform only first stage pretraining for our models", which sounds as if ALL experiments under section 2.3 (i.e. 2.3.1-2.3.4) use DAO-G with only stage I fine tuning. But it seems that this only holds for 2.3.1, whereas section 2.3.2 also deals with pretraining stage II.

Response 3.3-1:

You are right. We indeed used DAO-G (Stage I) in Section 2.3.1. We have removed the misleading sentence and clarified this point in Lines 198-203. Thank you for this helpful suggestion!

Comment 3.3-2

It would be better if section 2.3 contained a brief outline that explains the order of the following experiments, e.g. "first, we evaluate the performance gained by pretraining our model and the baselines on the deduplicated CrysDB, then we examine the effect of using a second pretraining stage II utilizing structures relaxed with DAO-P". Similarly, it might be helpful to add a sentence at the beginning of section 2.3.2 that briefly explains that now the whole setup (stage I+II) is tested in contrast to only stage I pretraining.

Response 3.3-2:

Thank you very much for this excellent suggestion. We agree that providing a brief outline greatly helps readers follow the logic and progression of the experiments.

Following the recommendation, we have added a concise outline in Section 2.3 to clarify the organization of the experimental results (Lines 198-203). Specifically, we now state that "In the following, we first evaluate the performance gains conferred by Stage-I pretraining on the deduplicated CrysDB for both our model and the baseline methods (Section 2.3.1). We then investigate the additional benefits of a second pretraining stage, which incorporates unstable structures relaxed by DAO-P (Section 2.3.2). Furthermore, we explore DAO-G's capability in generating polymorphic structures (Section 2.3.3) and demonstrate how energy-based guidance can further enhance the generation of structurally stable materials (Section 2.3.4)".

In addition, at the beginning of Section 2.3.2, we explicitly clarify that the full training pipeline (Stage I + Stage II) is evaluated in this subsection, in contrast to the Stage I–only setting studied in Section 2.3.1.

We believe these clarifications improves the readability and logical flow of Section 2.3, and we thank the reviewer again for this helpful and constructive suggestion.

Comment 3.3-3

Table 1 says Crysformer DiffCSP (DAO-G), albeit consisting only of stage I pretraining. The rest of the paper refers to DAO-G as using both pretraining stages, so maybe you should write (DAO-G stage I) in Table 1 or remove (DAO-G) altogether.

Response 3.3-3:

Thanks for your suggestion. we have now removed (DAO-G) in Table 1 to improve clarity and presentation.

Comment 3.4

Lines 383-384 read "First, we intentionally perturbed the experimental structures before relaxation to evaluate DAO-G's robustness" but the paragraph is dealing with DFT-based structure prediction. According to the supplements, the superconductor structures obtained with DAO-G are sampled from complete noise (not from noisy test structures). So how is the noise added to structures prior to relaxation with DFT connected to the robustness of DAO-G? We do not really understand this sentence.

Response 3.4:

Thank you for pointing out this ambiguity. We agree that the original wording could be

misleading, and we appreciate the opportunity to clarify the intent of this experiment.

Our goal in this paragraph was to contrast the robustness and practicality of DAO-G against DFT-based structure prediction when starting from highly uninformative initial conditions. For DAO-G, the generation process naturally samples structures from complete noise, as correctly noted by the reviewer. However, we found that DFT relaxations starting from complete noise generally fail to converge or lead to physically unreasonable structures, making a direct comparison infeasible.

To enable a meaningful comparison, we therefore adopted a more favorable initialization protocol for DFT: instead of starting from pure noise, we applied mild random perturbations to known experimental structures before relaxation. This setup provides DFT with substantially more prior structural information than is available to DAO-G, representing a better-case starting point for DFT.

As illustrated by the Cr_6Os_2 example (Figure 4g), even under this more favorable initialization, DFT remains significantly slower and converges to an inferior local minimum. In contrast, DAO-G, despite starting from complete noise, consistently produces structures with lower RMSE (0.0012 vs. 0.0410) and much higher efficiency (more than 2000 times faster).

We have revised the corresponding text (Lines 377-380) to explicitly state this motivation and avoid confusion. We hope this clarification underscores that the experiment demonstrates the robustness and practical advantage of DAO-G under less informative initial conditions.

Comment 3.5

In the mathematical definitions of the evaluation metrics, using M for the total number of generated samples for both MR and RMSE is a bit confusing, since RMSE is only computed for matched structures and not over the whole set of generated samples. Furthermore, the MR formula only works for the 1-shot case, not for 20-shot generation, as the same index is used for the set of generated structures and test structures. Please make the formulas more precise.

Response 3.5:

Thank you for this comment. You are correct that the RMSE is computed only over the successfully matched subset, not the full set, and we have revised Formulas 13 and 14 accordingly:

$$\mathrm{MR} = \frac{|\mathcal{I}|}{M}, \quad \mathcal{I} = \{i \in \{1,\ldots,M\} \mid \mathrm{StructureMatcher}(G_i, T_i) = \mathrm{True}\}, \tag{1}$$

$$\mathrm{RMSE} = \frac{1}{|\mathcal{I}|} \sum_{i \in \mathcal{I}} \Big(\frac{1}{\sqrt[3]{V_i/N_i}} \sqrt{\sum_{j=1}^{N_i} \|\mathbf{r}_{i,j} - \mathbf{r}'_{i,j}\|^2}\Big), \tag{2}$$

where, M is the total number of generated samples, G_i is the i-th generated structure, T_i is the corresponding i-th ground-truth structure, $\mathcal{I}$ denotes the subset of successfully matched data indices; V_i is the volume of the i-th crystal, N_i is its atom number, $\mathbf{r}_{i,j}$ and $\mathbf{r}'_{i,j}$ are the Cartesian coordinates of the j-th matched atom in G_i and Structure T_i, respectively, and $\|\cdot\|$ denotes the Euclidean distance.

Regarding 20-shot generation, for each target structure T_i, we generate 20 candidate structures. Each generated structure is first compared with G_i using the StructureMatcher. Among

the subset of candidates that are successfully matched, we select the one with the smallest structural error as G_i (i.e., $\arg\min_{k\in\{1,\ldots,20\}} \left(\frac{1}{\sqrt[3]{V_i/N_i}} \sqrt{\sum_{j=1}^{N_i} \|\mathbf{r}_{i,j} - \mathbf{r}'_{k,j}\|^2}\right)$). After determining G_i for all target structures in this manner, the Match Rate and RMSE are computed according to the above formulas.

We have clarified this distinction in Section 4.6 (Lines 659-664).

Comment 3.6

We still believe that Fig 3d should show the average change in energy instead of the sum. We don't really see a merrit in the current version of the graph since we neither know the number of samples per bin nor the average change in energy, therefore it does not tell much about the distribution of the data or the energy change. A large bar could stem from either a large number of samples or large changes in energies. If you would show the average instead, it would be clear by how much the systems have been improved on average during relaxation, depending on the initial energy.

Response 3.6:

Thank you for this constructive and insightful suggestion. We agree that plotting the sum of energy changes can conflate two factors: the number of samples per bin and the magnitude of energy reduction per structure, and therefore does not clearly convey how much each system improves on average during relaxation.

Following your recommendation, we have revised Fig. 3d (also shown in Figure R1 below) to report the average energy change within each bin, i.e., the total energy reduction normalized by the number of samples in that bin. We have also updated the corresponding description in Section 2.3.2 (Lines 238-240) to reflect this change. With this revised visualization, it becomes clearer that structures with higher initial instability (larger Ehull) experience a more pronounced average energy reduction during relaxation, which more directly and transparently demonstrates the effectiveness of our relaxation strategy.

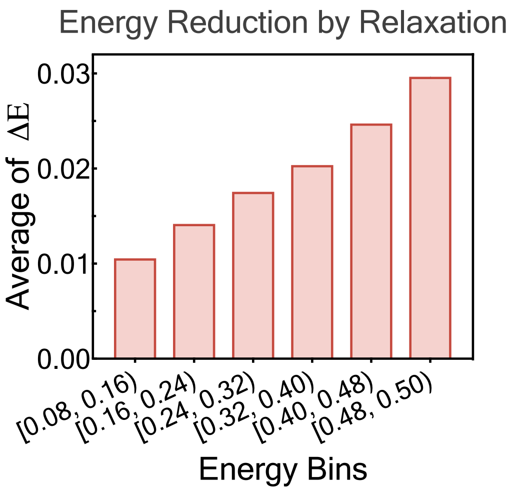

Figure R1: Averaged energy reduction across bins after dataset relaxation.

We believe this modification significantly improves the interpretability of the figure and better communicates the underlying distributional trend, and we thank the reviewer for helping us improve this aspect of the presentation.

References

[1] R. Flukiger, A. Paoli, and J. Muller, “Electronically ‘atypical’a 15-type compounds based on chromium and molybdenum,” *Solid State Communications*, vol. 14, no. 6, pp. 443–447, 1974.

Response to Reviewer #4

Comment 4.1

I co-reviewed this manuscript with one of the reviewers who provided the listed reports. This is part of the Nature Communications initiative to facilitate training in peer review and to provide appropriate recognition for Early Career Researchers who co-review manuscripts.

Response 4.1:

Thank you very much for taking the time to co-review our manuscript as part of *Nature Communications*' initiative to support early-career researchers in peer review. We sincerely appreciate the effort and thoughtful insights you and your co-reviewer have contributed. Your feedback has significantly strengthened the clarity, rigor, and overall quality of our work.

We have carefully addressed all the points raised in the review reports and revised the manuscript accordingly. We hope that the changes we have made adequately address your concerns and improve the manuscript to meet the journal's high standards. Thank you again for your valuable contribution to our paper.

Response to Reviewer #5

Comment 5.1

1.1 I agree with the authors response, that this clarification would suggest that there is little risk of data leakage. (there will be some unavoidable, small risk of this occurring, if there are errors in the results from structure matcher).
1.2 I agree that the authors have satisfactorily responded to the reviewer's comment. While I agree with the statement that Crysformer is improving the model performance. By my understanding, this is shown by comparison of DiffCSP-large with "Our Crysformer + Diffusion) and FlowMM-large and "Our Crysformer + Flow-Matching". If this is correct, it looks like using Crysformer is adding 1-3% to the performance across both of the match rates. Whereas, increasing the size of the training data does seem to have a greater impact on performance. I think that this has been reflected fairly in the revised text.
1.3 I think that this question has been answered, and the appropriate explanation added into section 4.
1.4 I am happy that this comment has been appropriately answered.
1.5 I am happy that this comment has been answered.
1.6 I thank the authors for their response to the question. Here, I find it surprising that there does not seem to be a systematic change in success rate with the number of polymorphs. Could the authors comment on this? Is it simply because of the small number of examples shown for tri and tetra morphs?

Response 5.1:

Thank you for your positive feedback on our revision. The paper has been significantly improved thanks to the thoughtful and valuable suggestions from all the reviewers.

Regarding Question 1.6, thank you for the insightful observation. In principle, the task should become more challenging as the number of polymorphs increases. We agree with the reviewer that the absence of a systematic trend in our results is likely due to the limited number of examples for tri- and tetramorphs. The limited sample size results in high variance in the estimated success rates, preventing strong conclusions about systematic behavior.

We have clarified these points in the revised manuscript (Lines 271-274) to better contextualize the observed results.

Comment 5.2

In the paragraph starting at line 375, the authors refer to conventional global optimization methods and refer to their computational cost. What the authors do not discuss here is that in many conventional CSP codes, they can be used with interatomic potentials, either classical, or machine -learned. Doing this can provide speed ups of a similar order of magnitude which the authors report here. Discussion of this does need to be included in the manuscript as well. For example the CSP codes FUSE, USPEX (already referred to in the paper) and AIRSS all can make routine use of fast interatomic potentials, and can be used either purely with potentials, or a combination of potentials and DFT. Related to this, I wonder, could DAO-P be used as an interatomic potential with a different CSP method such as those listed above?

Response 5.2:

Thank you for this insightful suggestion. You are correct that CSP codes such as USPEX, FUSE, and AIRSS can leverage fast interatomic potentials—either classical or machine-learned—to achieve significant acceleration. In practice, these methods are indirect CSP approaches, which first predict a potential energy surface and then optimize structures along its gradients. However, we would like to emphasize that these methods encounter a fundamental trade-off between accuracy and computational efficiency. For instance, while machine-learned potentials can expedite the CSP process, their performance strongly depends on the accuracy of the predicted potential; errors that accumulate during relaxation can lead to significant deviations in the final stable structure.

In contrast, our DAO-G directly learns the distribution of stable crystal structures, ensuring both competitive efficiency and high accuracy. We agree that the discussion raised by the reviewer is important and have incorporated relevant points in the manuscript (Lines 385-389).

Furthermore, as noted by the reviewer, DAO-P could in principle be integrated as an interatomic potential into conventional CSP codes via an appropriate interface. In fact, DAO-P already serves as energy guidance during the sampling process of DAO-G, inherently leveraging its capabilities as a potential predictor. This internal integration ensures a tight coupling between the energy model and the structural generator, which allows DAO-G to benefit from predictive energy guidance while maintaining the direct learning of structural distributions.

Comment 5.3

It would be useful for readers, if when summarizing the paper in the discussion section (line 396 onwards) to discuss the importance of the size of available training data, and it's impact in model performance. This is a significant contribution of the paper, is the assembled CrysDB database.

Response 5.3:

We appreciate this insightful suggestion. We agree that highlighting the impact of training data size strengthens the discussion.

We have further emphasized that "the majority of existing CSP models are trained on relatively small datasets such as MP-20 and MPTS-52, which limits their ability to learn diverse structural patterns and generalize to more complex systems. By contrast, the large-scale, high-quality CrysDB dataset assembled in this work enables effective pretraining, providing the model with broader structural knowledge and significantly improving its generative performance and robustness" (Lines 405-409).

Comment 5.4

Given the use of a model to predict energies of crystals, it would be useful to refer to the paper by Chang Liu et al (https://www.nature.com/articles/s41524-024-01471-8), or other papers where energy is a learned property which is then used to aid in the prediction of structure.

Response 5.4:

Thank you for recommending this excellent paper, which is closely related to our work and has provided valuable inspiration. We have cited and discussed it in the Introduction (Lines 43-46).

Responses to Reviewers' Comments for Manuscript:

Siamese Foundation Models for Crystal Structure Prediction

by
Liming Wu (Renmin University of China)
Wenbing Huang (Renmin University of China)
Rui Jiao (Tsinghua University)
Jianxing Huang (Huawei Technologies)
Liwei Liu (Huawei Technologies)
Yipeng Zhou (Huawei Technologies)
Hao Sun (Renmin University of China)
Yang Liu (Tsinghua University)
Fuchun Sun (Tsinghua University)
Yuxiang Ren (Nanjing University)
Ji-Rong Wen (Renmin University of China)

Contents

Response to Reviewer 3 1

Response to Reviewer 4 2

Response to Reviewer 5 3

Response to Reviewer #3

Comment 3.1

As in the first round of rebuttal, we want to thank the authors for taking the reviewer's comments very seriously and for their detailed answers. The authors have now clearly documented their choice of hyperparameters, ensuring the reproducibility of their work and improving the interpretability of the presented results. Furthermore, they have updated the experiments on superconductors, removing the results on CsV3Sb5 (which was affected by data leakage) and adding a study on Cr6Os2. The additional changes have further strengthened the presented results and improved the quality of the manuscript. Overall, our concerns regarding scientific best practices have been addressed and we believe that the manuscript is now suitable for publication, presenting valuable insights on AI guided materials discovery. We still believe that the technical contributions (creating and utilizing a large-scale data set for pretraining + evaluation of the method on an interesting application example) better fit in a journal that is specialized on computational materials science (e.g. npj Computational Materials). However, due to the high relevance of the application domain and the extent of the presented studies, we also consider it (borderline) in-scope for Nature Communications.

Response 3.1:

We are pleased that our responses and modifications have addressed your concerns about scientific best practices. Your feedback has been invaluable in helping us improve both the clarity and robustness of the manuscript, and we are glad that you now find it suitable for publication and believe it provides meaningful insights for AI-guided materials discovery. Thank you very much for your positive and encouraging assessment of our revised manuscript.

Response to Reviewer #4

Comment 4.1

I co-reviewed this manuscript with one of the reviewers who provided the listed reports. This is part of the Nature Communications initiative to facilitate training in peer review and to provide appropriate recognition for Early Career Researchers who co-review manuscripts.

Response 4.1:

We are deeply grateful for the time you dedicated to co-reviewing our manuscript under *Nature Communications*' early-career researcher initiative. We sincerely appreciate the thoughtful insights provided by both you and your co-reviewer. Your feedback has been instrumental in enhancing the clarity, rigor, and overall quality of our work.

Response to Reviewer #5

Comment 5.1

In the sentence starting on line 387, the authors write:
"In contrast, our DAO-G directly learns the distribution of stable crystal structures, ensuring both competitive efficiency and high accuracy."
This sentence, does not quite read correctly to me. The existing CSP methods, when using interatomic potentials can both use foundation level ML models as well as potentials trained for the specific system of interest. This means that in practice, the potentials that these codes can use are also trained on a wide range of crystal structures. e.g. When using MACE foundation potentials. And so potentially, this work is likely to have similar advantages and drawbacks as these potential models.
If left in the paper, this statement should be more in line with previous work, not in contrast. One thing that the authors can highlight differences in the training data which is available to each of their models.

Response 5.1:

Thank you for your insightful comment regarding the sentence on line 387. We agree that the original phrasing could be misinterpreted as overlooking existing methods that also utilize models trained on diverse crystal data, such as MACE foundation potentials. To address this, we have revised the sentence to more accurately reflect the relationship between our approach and existing methods. The revised text now reads:

"While existing methods often leverage interatomic potentials trained on extensive crystal data, our DAO-G offers a complementary approach by directly learning the distribution of stable structures, aiming for an optimal balance of efficiency and accuracy."

This revision acknowledges the strengths of existing approaches while clearly articulating the distinct methodology and contribution of our work. We believe this provides a more balanced and accurate comparison. Thank you for helping us improve the clarity and precision of our manuscript.
